# The efficacy and safety of proprotein convertase subtilisin/kexin type 9 (PCSK9) inhibitors combined with statins in patients with hypercholesterolemia: a network meta-analysis

**DOI:** 10.3389/fcvm.2024.1454918

**Published:** 2024-09-25

**Authors:** Dong Liu, Jin Zhang, Xiaoyu Zhang, Fengli Jiang, Yiping Wu, Beibei Yang, Xinghuan Li, Xiongxiong Fan, Han Li, Yu Sun, Ruijie Gou, Xinyu Wang

**Affiliations:** Clinical Pharmacy Office, Baoji Central Hospital, Baoji, Shaanxi, China

**Keywords:** hypercholesterolemia, PCSK9 inhibitors, low-density lipoprotein cholesterol, apolipoprotein B, lipoprotein (a), meta-analysis

## Abstract

**Background:**

In recent years, the position of PCSK9 inhibitors as adjuvant therapy to statins in guidelines has further improved. However, there remained a dearth of direct comparative studies among different PCSK9 inhibitors. Therefore, this study aimed to conduct a network meta-analysis to evaluate the efficacy and safety of different PCSK9 inhibitors combined with statins.

**Methods:**

A comprehensive literature search was conducted from the study's inception to 12 November 2023, encompassing multiple online databases including PubMed, Embase, Cochrane Central, Web of Science, and ClinicalTrials.gov to obtain relevant randomized controlled trials. Frequentist network meta-analysis was employed to compare the efficacy and safety of different PCSK9 inhibitors. The efficacy endpoints were low-density lipoprotein cholesterol (LDL-C), apolipoprotein B (ApoB), and lipoprotein (a) (Lp(a)). The safety endpoints were any adverse events (AE), severe adverse events (SAE), AE leading to treatment discontinuation, and injection-site reaction.

**Results:**

Compared with placebo and ezetimibe, all PCSK9 inhibitors demonstrated significant reductions in LDL-C levels. Notably, evolocumab exhibited the most pronounced effect with a treatment difference of −63.67% (−68.47% to −58.87%) compared with placebo. Regarding dosage selection for evolocumab, the regimen of 140 mg Q2W (−69.13%, −74.55% to −63.72%) was superior to 420 mg QM (−61.51%, −65.97% to −57.05%). Based on rankings and *P*-scores analysis, tafolecimab 150 mg Q2W demonstrated superior efficacy in reducing ApoB levels (−61.70%, −84.38% to −39.02%) and Lp(a) levels (−43%, 30%, −68%, 81% to −17%, 79%). Furthermore, the safety profile of PCSK9 inhibitors was favorable with no increase in the incidence of AE, SAE, or AE leading to treatment discontinuation; however, alirocumab, inclisiran, and tafolecimab may potentially entail a potential risk associated with injection-site reactions.

**Conclusion:**

Compared with placebo and ezetimibe, PCSK9 inhibitors can significantly reduce LDL-C, ApoB, and Lp(a) when combined with statins to treat hypercholesterolemia. Furthermore, PCSK9 inhibitors and ezetimibe exhibit similar safety profiles.

**Systematic Review Registration:**

[PROSPERO], identifier [CRD42023490506].

## 1 Introduction

Dyslipidemia refers to alterations to the plasma lipid profile, with hypercholesterolemia being the predominant manifestation of dyslipidemia (1). Elevated levels of low-density lipoprotein cholesterol (LDL-C) are closely associated with an increased risk of cardiovascular disease and are considered a prominent risk factor for its development (2, 3). According to statistics, approximately 4.4 million deaths were associated with high levels of L DL-C in 2019 (1). Apolipoprotein B (ApoB) serves as the principal apolipoprotein of low-density lipoprotein, and several studies have found it to be a significant biomarker and predictor for hypercholesterolemia (4–6). Furthermore, lipoprotein (a) (Lp(a)) has also been proven as a potential pathogenic risk factor for cardiovascular disease and exhibited a certain degree of resistance to therapeutic lowering with statins (7–9).

Statins have long been widely used as cornerstone drugs in lipid-lowering therapies. However, despite receiving the maximum tolerated dose of statins to certain patients, they still failed to attain the expected LDL-C levels (10, 11). Concurrently, certain patients exhibit intolerance towards high-intensity statins (12, 13). Therefore, according to the 2018 American Heart Association/American College of Cardiology (AHA/ACC) guidelines and the 2019 European Society of Cardiology/European Atherosclerosis Society (ESC/EAS) guidelines, the addition of ezetimibe or PCSK9 inhibitor could be considered an adjunctive therapy to further attenuate LDL-C levels (14, 15).

PCSK9 inhibitors include monoclonal antibodies or small interfering RNA (siRNA) that exert their mechanism of action by inhibiting the activity or synthesis of PCSK9, thereby upregulating the expression of low-density lipoprotein receptor (LDLR) levels on the surface of liver cells, facilitating liver metabolism and clearance of LDL-C from plasma, resulting in a decrease in LDL-C levels (16–18). When statins reduce LDL-C levels, their negative feedback regulation triggers an increase in the expression and secretion of PCSK9, thereby attenuating its efficacy in lowering LDL cholesterol (19, 20). Consequently, combining PCSK9 inhibitors with statins emerges as a promising therapeutic strategy. The PCSK9 inhibitors including alirocumab, evolocumab, and inclisiran, received marketing approval from the Food and Drug Administration (FDA) in 2015 and 2021, and have been widely utilized in clinical practice (21–24). Additionally, tafolecimab received market approval from the National Medical Products Administration (NMPA) in August 2023. Compared with placebo, it can reduce LDL-C levels by approximately 57%–65%, maintain long-term treatment efficacy, and exhibit strong lipid-lowering abilities for ApoB and Lp(a) (25–27).

Given the absence of direct comparisons among different PCSK9 inhibitors, a network meta-analysis is commonly employed to comprehensively evaluate their efficacy and safety by synthesizing both direct and indirect evidence. However, previous systematic reviews and meta-analyses have predominantly focused on alirocumab, evolocumab, and inclisiran. Building upon this foundation, our study encompassed clinical trials pertaining to the latest approved tafolecimab and further compared the frequently-used clinical dosages of four medications to assess the efficacy and safety of different PCSK9 inhibitors in combination with statins for treating patients with hypercholesterolemia.

## 2 Methods

This network meta-analysis was reported in accordance with PRISMA and was registered with PROSPERO (CRD42023490506).

### 2.1 Data sources and search strategy

Several online databases were searched from the study's inception to 12 November 2023, including PubMed, Embase, Cochrane CENTRAL, Web of Science, and ClinicalTrials.gov, by applying the following medical subject heading (MeSH) search terms and keywords: “hypercholesterolemia”, “alirocumab”, “evolocumab”, “inclisiran”, “tafolecimab”, and “randomized controlled trials”, without language restrictions. The details of the search strategy conducted are presented in Supplementary Table S1.

### 2.2 Selection criteria

The studies were included if they met the following criteria: (1) the study population should be adult patients (age ≥ 18) who have hypercholesterolemia; (2) the intervention group used PCSK9 inhibitions and the control group was given a placebo or ezetimibe; (3) treatment should be based on therapy with statin background therapy; (4) the study was a phase 3 RCTs; (5) the study reported the percentage change from baseline in LDL-C, ApoB, or Lp(a).

The exclusion criteria were as follows: (1) the results have not been published or available data cannot be obtained; (2) duplicated reports; (3) conference articles, letters, reviews, commentaries, and case reports.

### 2.3 Data extraction and outcome assessments

EndNoteX9 was used to manage the literature, and all retrieved studies were preliminarily screened by two reviewers (XW and JZ) based on the established inclusion and exclusion criteria. Subsequently, the full texts of the literature were further read to determine which to be included in this study. The following information was extracted or calculated: trial name, first author's name, year of publication, number of patients, characteristics of patients (age, gender, diabetes mellitus, hypertension, and lipid profiles at baseline), treatment regimens, and duration of follow-up, and efficacy and safety endpoint. The process of screening and extraction was independently completed by two reviewers (XW and JZ), and disagreements were resolved through communication and discussion or evaluation by a third reviewer (FJ), ultimately reaching a consensus.

The quality of all included trial studies was critically assessed independently by two reviewers (XW and JZ) according to the Cochrane risk of bias tool, and they reached a consensus by consulting the third independent reviewer (FJ). The main assessment content included random sequence generation, allocation concealment, blinding, incomplete outcome data, selective reporting, and other biases. The risk assessment results were divided into three categories: high, low, and unclear, and the result was recorded using Review Manager software.

### 2.4 Statistical analysis

The statistical analysis of the data was performed using the “netmeta” package in R (4.0.1) software to compare the efficacy and safety of different PCSK9 inhibitors in the frequentist framework. Mean difference (MD) along with its 95% confidence interval (95% CI) were employed to represent continuous variables, while dichotomous variables were represented by risk ratio (RR) and its corresponding 95% CI. Under the consistency model, direct and indirect evidence were integrated using a random effects model. The consistency of treatment effects between studies with different sets of treatments was evaluated using the design-by-treatment interaction model, and the node-splitting method was employed to examine the differences between direct and indirect evidence. The *P*-score was calculated to rank treatments. The funnel plot was used to assess publication bias. If more than 10 trials were included, the possibility of publication bias was evaluated through the Egger regression test and Begg's rank test. Statistical heterogeneity was evaluated by *I*^2^ test. The *I*^2^ values of 25%, 50%, and 75% corresponded to mild, moderate, and severe heterogeneity, respectively. Utilizing a random effects model to estimate the combined effect size, while partially correcting for meta-analysis heterogeneity, enhances the precision of the CI. Finally, sensitivity analysis was performed by excluding specific research conditions: trials in familial hypercholesterolemia (FH) patients, baseline LDL-C level >130 mg/dl, and follow-up duration <24 weeks.

## 3 Results

### 3.1 Study selection and characteristics

A total of 2,622 articles were retrieved from databases and clinical trial registration databases. After removing duplicates and screening titles and abstracts, a preliminary selection was made with 55 articles. Subsequently, the full texts of these articles underwent meticulous review, resulting in the final inclusion of 25 articles that met the predetermined criteria. The PRISMA flowchart illustrating the screening process is presented in Supplementary Figure S1.

This network meta-analysis included a total of 26 trials, involving 16,510 participants, with sample sizes ranging from 107 to 2,341 participants; the mean age varied between 49.0 and 66.1 years old; the proportion of female participants ranged from 17.6% to 57.3%. Among 26 studies, 13 were based on alirocumab involving 6,723 patients, and 7 studies were based on evolocumab involving 5,062 patients. Additionally, there were 3 studies each for inclisiran and tafolecimab, encompassing 3,660 and 1,065 patients respectively. The included clinical trials studied four different doses of PCSK9 inhibitors, encompassing a total of 11 treatment regimens, including alirocumab 75 mg Q2W, 150 mg Q2W, 300 mg QM, and evolocumab 140 mg Q2W, 420 mg QM, and inclisiran 300 mg day 1, day 90, and every 6 months thereafter, and tafolecimab 150 mg Q2W, 450 mg Q4W, 600 mg Q6W vs. either ezetimibe or placebo. The characteristics of the included studies are reported in Table 1.

**Table 1:** Characteristics of included studies.

No	Author	Published year	Trail	Register number	Total patients	Age (mean)	Women (%)	DM (%)	HP (%)	Mean LDL-C (mg/dl)	Administration of PCSK9 modulators	Follow up (weeks)
1	Bays H	2015	ODYSSEY OPTIONS I	NCT01730040	206	64.0	36.4	50.0	78.7	104.9	Alirocumab 75 mg Q2W	24
2	Farnier M	2016	ODYSSEY OPTIONS II	NCT01730053	204	60.9	43.1	39.7	71	111.8	Alirocumab 75 mg Q2W	24
3	Kereiakes DJ	2015	ODYSSEY COMBO I	NCT01644175	316	63.0	34.2	43.0	/	103.1	Alirocumab 75 mg Q2W	52
4	Cannon CP/El Shahawy M	2015/2017	ODYSSEY COMBO II	NCT01644188	720	61.6	26.4	31.0	/	108.3	Alirocumab 75 mg Q2W	104
5	Kastelein JJP	2015	ODYSSEY FH I	NCT01623115	486	51.9	43.6	11.7	43.2	144.6	Alirocumab 75 mg Q2W	78
			ODYSSEY FH II	NCT01709500	249	53.2	47.4	4.0	32.5	134.4	Alirocumab 75 mg Q2W	78
6	Robinson JG	2015	ODYSSEY LONG TERM	NCT01507831	2,341	60.5	37.8	34.6	/	122.3	Alirocumab 150 mg Q2W	78
7	Roth EM	2016	ODYSSEY CHOICE I	NCT01926782	547	61.3	37.5	30.7	/	113.1	Alirocumab 75 mg Q2W/300 mg Q4W	48
8	Ginsberg HN	2016	ODYSSEY HIGH FH	NCT01617655	107	51.0	46.7	14.0	57.0	198.7	Alirocumab 150 mg Q2W	78
9	Teramoto	2016	ODYSSEY JAPAN	NCT02107898	216	60.8	39.4	68.5	/	141.2	Alirocumab 75 mg Q2W	52
10	Leiter LA	2017	ODYSSEY DM-INSULIN	NCT02585778	517	60.3	44.9	100	/	114.3	Alirocumab 75 mg Q2W	24
11	Koh KK	2018	ODYSSEY KT	NCT02289963	199	60.7	17.6	35.2	/	98.15	Alirocumab 75 mg Q2W	24
12	Han Y	2019	ODYSSEY EAST	NCT02715726	615	58.6	25.0	27.5	60.0	110.8	Alirocumab 75 mg Q2W	24
13	Robinson JG	2014	LAPLACE-2	NCT01763866	1,896	60.1	45.8	15.5	/	108.9	Evolocumab 140 mg Q2W/420 mg Q4W	12
14	Blom DJ	2014	DESCARTES	NCT01516879	790	56.8	51.9	12.8	49.5	104.5	Evolocumab 420 mg Q4W	52
15	Raal FJ	2015	RUTHERFORD-2	NCT01763918	329	51.0	42.2	/	/	154.7	Evolocumab 140 mg Q2W/420 mg Q4W	12
16	Kiyosue A	2016	YUKAWA-2	NCT01953328	404	61.5	39.6	48.8	73.5	106.0	Evolocumab 140 mg Q2W/420 mg Q4W	12
17	Rosenson RS	2019	BANTING	NCT02739984	421	62.4	43.9	100	86.9	109.6	Evolocumab 420 mg Q4W	12
18	Lorenzatti AJ	2019	BERSON	NCT02662569	981	61.3	57.3	100	73.1	92.8	Evolocumab 140 mg Q2W/420 mg QM	12
19	Tan H	2023	HUA TUO	NCT03433755	241	60.2	32.3	16.4	62.2	116.8	Evolocumab 140 mg Q2W/420 mg QM	12
20	Raal FJ	2020	ORION-9	NCT03397121	482	56.0	52.9	10.0	42.1	153.1	Inclisiran 300 mg day 1, day 90, and every 6 months thereafter	78
21	Raal KK	2020	ORION-10	NCT03399370	1,561	66.1	30.6	45.0	90.6	104.7	Inclisiran 300 mg day 1, day 90, and every 6 months thereafter	78
			ORION-11	NCT03400800	1,617	64.8	28.3	35.1	80.5	105.5	Inclisiran 300 mg day 1, day 90, and every 6 months thereafter	78
22	Huo Y	2023	CREDIT-1	NCT04289285	614	57.4	34.0	36.6	75.6	110.1	Tafolecimab 450 mg Q4W/600 mg Q6W	48
23	Meng C	2023	CREDIT-2	NCT04179669	148	49.0	48.0	/	/	146.8	Tafolecimab 150 mg Q2W/450 mg Q4W	12
24	Qi L	2023	CREDIT-4	NCT04709536	303	56.8	31.0	34.3	/	118.3	Tafolecimab 450 mg Q4W	12

DM, type 2 diabetes mellitus; HP, hypertension.

### 3.2 Risk-of-bias assessment

The risk of bias in all included studies was assessed using the Cochrane risk-of-bias assessment tool across seven domains. The findings revealed that allocation consideration emerged as the most common category associated with potential bias risk among the studies. Most randomized controlled trials demonstrated low or unclear bias risk, with only one trial being rated as having a high bias risk due to incomplete outcome data. Detailed information regarding the Cochrane bias risk assessment for each study can be found in Supplementary Figures S2, S3.

### 3.3 Efficacy endpoints

#### 3.3.1 Low-density lipoprotein cholesterol

**Figure 1:**
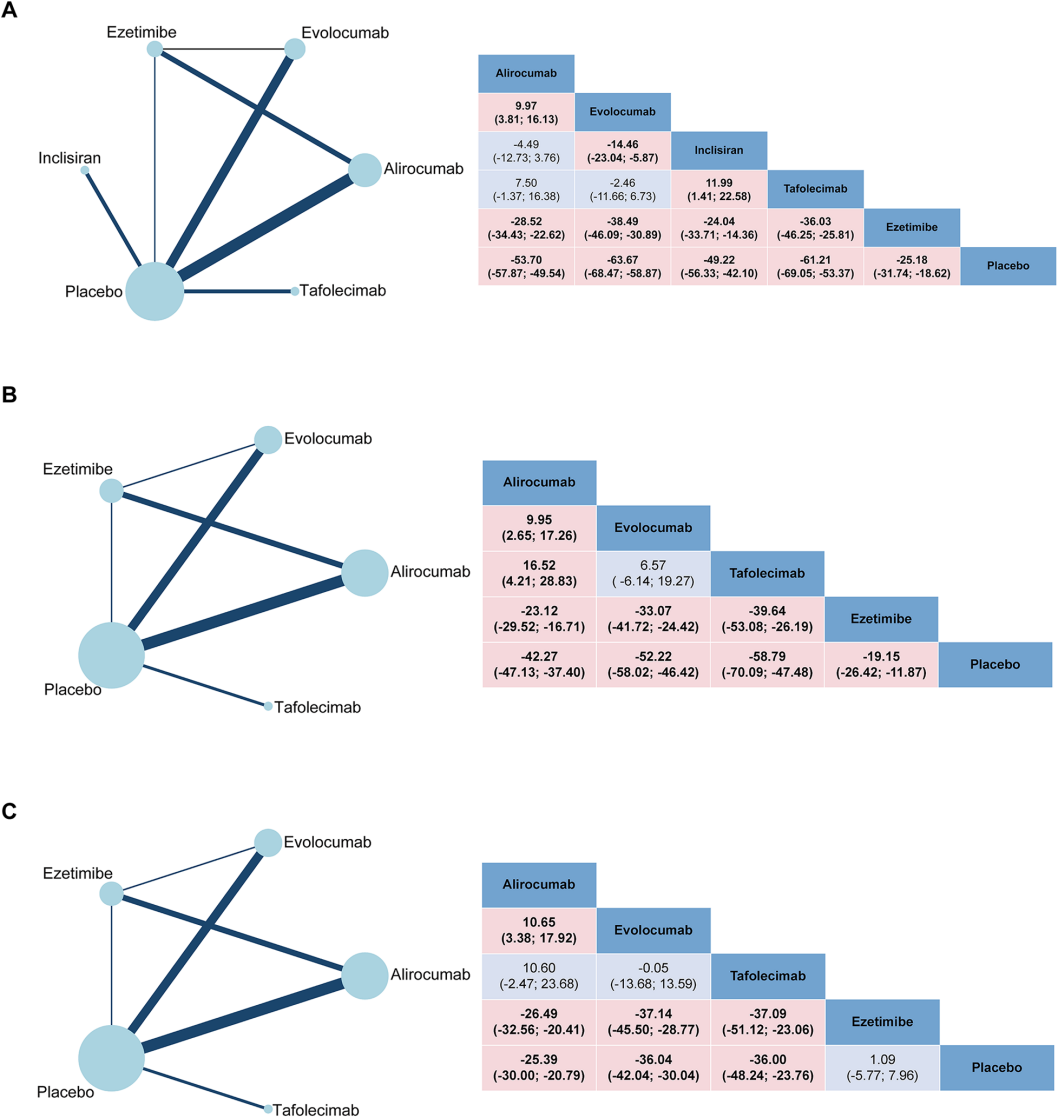
Network geometry and league table of **(A)** LDL-C; **(B)** apoB and **(C)** Lp(a). The left column showed the corresponding network geometry of each outcome. The right column showed the corresponding league table of each outcome. The result is represented in mean difference (MD) and 95% confidence interval (CI). Significant pairwise comparisons are highlighted.

The network plots and the results of network meta-analysis for LDL-C reduction are presented in Figure 1A. Compared with placebo and ezetimibe, all four PCSK9 inhibitors exhibited significant reductions in levels of LDL-C, with evolocumab (MD −63.67%, 95%CI −68.47% to −58.87%) and tafolecimab (−61.21%, −69.05% to −53.37%) demonstrating similar LDL-C level reduction effects (Supplementary Figure S4A). Evolocumab 140 mg Q2W had the best performance in reducing LDL-C levels, with the treatment difference being −69.13% (−74.55% to −63.72%) compared with placebo, followed by tafolecimab 450 mg QM (−63.94%, −71.36% to −56.51%) and evolocumab 420 mg QM (−61.51%, −65.97% to −57.05%) (Supplementary Figure S10A). There was no significant difference in LDL-C reduction observed between different doses of alirocumab and tafolecimab. However, a significant decrease in LDL-C levels was observed with evolocumab 140 mg Q2W (−7.62%, −14.58% to −0.66%) compared with evolocumab 420 mg QM when evaluating different doses of evolocumab (Supplementary Figure S8A).

#### 3.3.2 Apolipoprotein B

The network plots and the results of network meta-analysis for ApoB reduction are presented in Figure 1B. Compared with placebo and ezetimibe, all three PCSK9 inhibitors demonstrated a significant impact on ApoB reduction (Inclisiran was excluded due to lack of reported data on ApoB), with tafolecimab (−58.79%, −70.09% to −47.48%) exhibited a greater decrease in ApoB levels compared with evolocumab (−52.22%, −58.02% to −46.42%) and alirocumab (−42.27%, −47.13% to −37.40%) (Supplementary Figure S4B). Among the various treatment strategies, tafolecimab 150 mg Q2W (−61.70%, −84.38% to −39.02%) ranked first, followed by tafolecimab 450 mg QM (−58.05%, −67.94% to −48.16%) as the second most effective option (Supplementary Figure S10B). No statistically significant differences were observed in the reduction of ApoB levels among the three PCSK9 inhibitors at different doses (Supplementary Figure S8B).

#### 3.3.3 Lipoprotein (a)

The network plots and the results of network meta-analysis for Lp(a) reduction are presented in Figure 1C. Compared with placebo and ezetimibe, all PCSK9 inhibitors showed superior efficacy in reducing Lp(a) levels [Inclisiran was excluded due to the lack of reported data on Lp(a)], among which evolocumab (−36.04%, −42.04% to −30.04%) and tafolecimab (−36.00%, −48.24% to −23.76%) had similar effects in reducing Lp(a) levels (Supplementary Figure S4C). There was no significant difference in Lp(a) reduction between ezetimibe and placebo. Tafolecimab 150 mg Q2W (−43.30%, −68.81% to −17.79%) remained the optimal treatment strategy for reducing Lp(a) levels compared with placebo across all treatment strategies (Supplementary Figure S10C).

### 3.4 Safety endpoints

**Figure 2:**
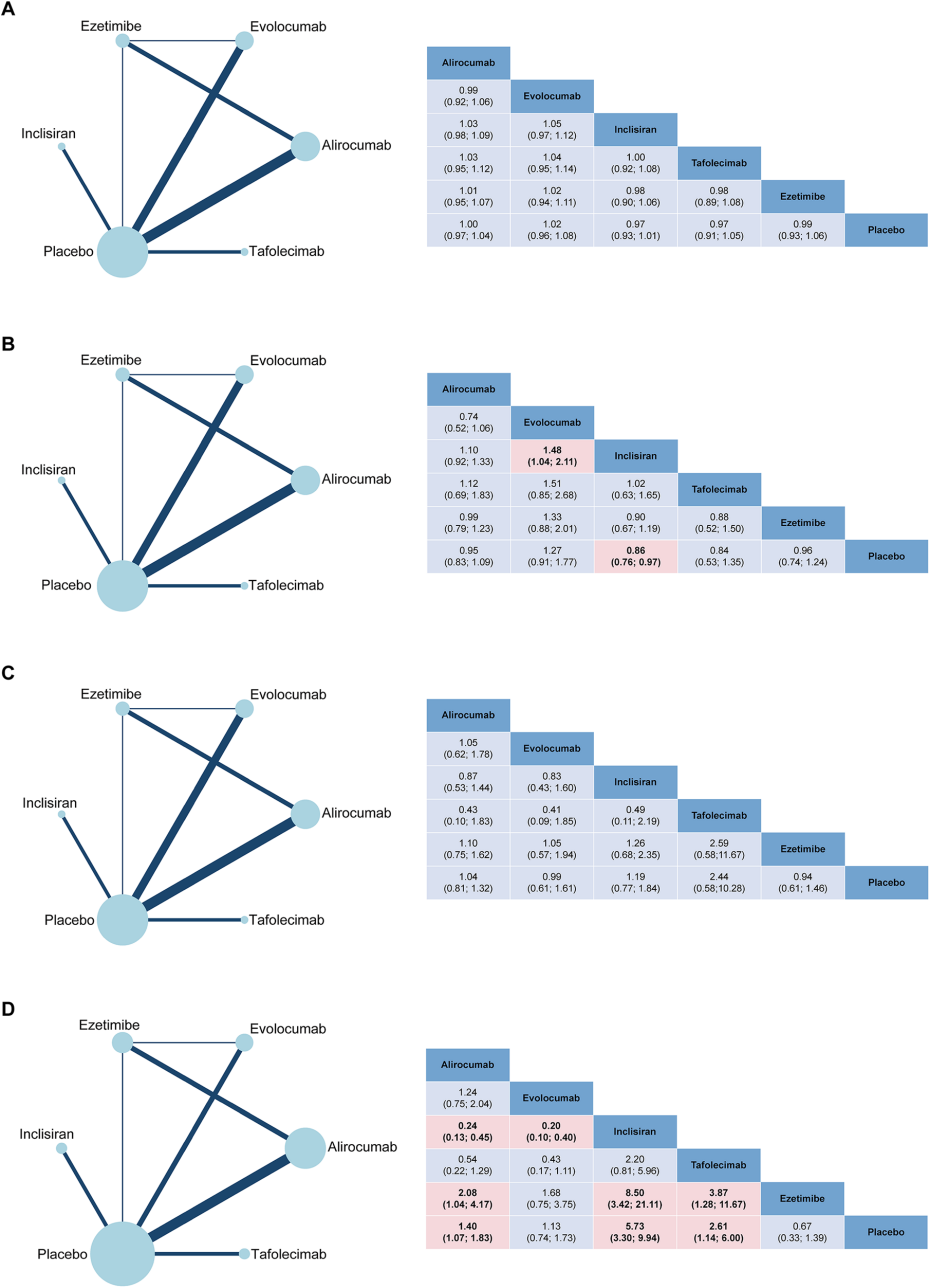
Network geometry and league table of **(A)** AE; **(B)** SAE; **(C)** AE leading to treatment discontinuation and **(D)** injection-site reaction. The left column showed the corresponding network geometry of each outcome. The right column showed the corresponding league table of each outcome. The result is represented in risk ratio (RR) and 95% CI. Significant pairwise comparisons are highlighted.

The network plots and the results of network meta-analysis for Safety endpoints are presented in Figure 2. There were no statistically significant differences in the risk of any adverse events (AE) between alirocumab (RR 1.00, 95% CI 0.97–1.04), evolocumab (1.02, 0.96–1.08), inclisiran (0.97, 0.93–1.01), tafolecimab (0.97, 0.91–1.05), or ezetimibe (0.99, 0.93–1.06) and placebo except for the QM doses of tafolecimab (Supplementary Figure S5A, S11A). Tafolecimab 450 mg QM (0.91, 0.84–0.99) was associated with reductions in any AE compared with placebo. In addition, when comparing different doses of the same drug, there was a significant reduction in the risk of AE observed with alirocumab 150 mg Q2W (0.89, 0.80–0.99) compared with 300 mg QM. Similarly, tafolecimab 450 mg QM (0.87, 0.76–0.99) demonstrated a lower potential risk of AE compared with 600 mg Q6W. The administration of inclisiran (0.86, 0.76–0.97) was associated with reductions in severe adverse events (SAE) compared with placebo (Supplementary Figure S5B). Similar results were observed in the comparison of different doses (0.83, 0.70–0.99) (Supplementary Figure S11B).

None of alirocumab (1.04, 0.81–1.32), evolocumab (0.99, 0.61–1.61), inclisiran (1,19, 0.77–1.84), tafolecimab (2.44, 0.58–10.28) or ezetimibe (0.94, 0.61–1.46) were associated with an increase of AE leading to treatment discontinuation as compared with placebo (Supplementary Figure S5C). Different doses of PCSK9 inhibitors were also unrelated to AE leading to treatment discontinuation (Supplementary Figure S11C). The use of inclisiran (5.73, 3.30–9.94), tafolecimab (2.61, 1.14–6.00), and alirocumab (1.40, 1.07–1.83) were associated with increased risk in injection-site reaction (Supplementary Figure S5D). In the comparison between different doses of PSCK9 inhibitors and placebo, inclisiran 300 mg (5.73, 3.30–9.94) significantly increased the risk of injection-site reaction (Supplementary Figure S11D).

### 3.5 Inconsistency assessment, publication bias and sensitivity analyses

The design-by-treatment interaction model and the node-splitting method were employed in the inconsistency test, which revealed no evidence of inconsistency. The funnel plots of LDL-C and ApoB showed possible publication bias, and the asymmetric source may come from larger studies. For the assessment of publication bias for Lp(a) and safety endpoints from PCSK9 inhibitors, no significant publication bias was found. However, due to the lack of direct head-to-head research, these outcome results may exhibit some inaccuracies and heterogeneity. In addition, the study included as many as 11 treatment options with dosing intervals ranging from 2 weeks to 6 months, potentially leading to clinical heterogeneity due to multiple intervention measures. To exclude potential sources of heterogeneity, we performed sensitivity analysis by excluding specific studies. The results indicated that there was no significant change in the ranking of each PCSK9 inhibitor before and after the sensitivity analyses, which were consistent with the primary results. This indicates that the meta-analysis results possess a certain degree of robustness and enhance the credibility of our analytical outcomes.

## 4 Discussion

The good lipid-lowering efficacy of PCSK9 inhibitors has been demonstrated in numerous meta-analyses. Traditional meta-analyses predominantly focused on comparing PCSK9 inhibitors as a singular class, or solely emphasized different types of PCSK9 inhibitors while overlooking dosage considerations. The latest two network meta-analyses have further evaluated the therapeutic variances among different doses of PCSK9 inhibitors. For instance, Toth et al. identified evolocumab 140 mg Q2W/420 mg QM and alirocumab 150 mg Q2W as the most efficacious non-statin drugs, potentially enabling a larger proportion of patients to achieve LDL-C levels in accordance with current guidelines (28). Additionally, another study highlighted that evolocumab 140 mg Q2W exhibited superior effectiveness compared with other treatment strategies in reducing LDL-C and ApoB levels (29). Unlike previous studies that only compared three PCSK9 inhibitors with statin and ezetimibe, our study also included tafolecimab to comprehensively assess the lipid-lowering ability of PCSK9 inhibitors based on the latest clinical evidence. Consistent with prior research findings, our study further substantiates that evolocumab 140 mg Q2W demonstrates the greatest reduction in LDL-C level.

Additionally, in addition to LDL-C, we selected ApoB and Lp(a) as efficacy endpoints. According to the 2019 ESC/EAS guidelines, ApoB was found to be a more accurate measure of atherosclerotic cardiovascular disease risk and lipid-lowering treatment adequacy compared with LDL-C (15). Moreover, its measurement results were also more precise than those of LDL-C (15, 30). The Lp(a) as a potential target for lipid-lowering therapy has gained prominence in recent years, supported by strong evidence indicating that elevated levels of Lp(a) can increase the risk of cardiovascular events even with effective LDL-C control (31, 32). However, commonly used LDL-C lowering drugs have limited or no impact on Lp(a) (33, 34). In contrast, PCSK9 inhibitors have shown the ability to reduce Lp(a) levels to a certain extent, and the precise mechanism underlying this effect requires further investigation, which may be related to PCSK9 inhibitors promoting an increase in Lp(a) catabolism or a decrease in production (35–37). A direct comparative study demonstrated that PCSK9 inhibitors achieved a significant 26.7% reduction in Lp(a) levels compared with the control group (38). Another network meta-analysis revealed that evolocumab 140 mg Q2W exhibited the most pronounced therapeutic efficacy, which aligned with our research findings (39). In summary, PCSK9 inhibitors hold promise as therapeutic drugs for reducing plasma Lp (a) levels.

In terms of safety, our meta-analysis of adverse reactions indicated that there was an increased risk of AE at high doses compared with medium doses for both alirocumab and tafolecimab. Both two demonstrated effective control over lipid levels at low to moderate doses. The reason for this result may be that multiple low to moderate-dose administrations help maintain stable drug concentrations, avoid excessive fluctuations in blood drug concentrations, and reduce the potential side effects or toxicity caused by a single large dose. Moreover, due to differences in individual metabolism and excretion abilities, drugs accumulate in the body, making them more prone to safety issues at high doses. As the dose increases, the interaction between the drug and biomolecules increases or reaches saturation with the target, and additional doses not only fail to increase efficacy but may also cause adverse reactions. Therefore, when selecting between different recommended doses in the instruction manual, if the difference in benefit levels is not significant, opting for a lower or moderate dose may help reduce the occurrence of adverse reactions. However, the literature currently lacks reports on whether the incidence of adverse reactions exhibits dose dependence. Given the overall low incidence rate of AE associated with PCSK9 inhibitors, cautious consideration should be exercised until sufficient evidence and explanations are available. Furthermore, in comparison to the other three drugs, Inclsiran exhibits a significantly higher risk of injection site reactions. This disparity may be attributed to the fact that alirocumab, evolocumab, and tafolecimab are fully humanized monoclonal antibodies that usually have good tolerance. However, inclisiran is a siRNA that exhibits differences in physicochemical properties, which may lead to injection site reactions. It is important to note that most adverse events occurring at the injection site of inclisiran are mild or moderate in nature and self-limiting, resolving without intervention. Consequently, overall safety profiles remain satisfactory across all four drugs.

The strength of this study lies in the inclusion of the latest marketed tafolecimab, which updated the intervention strategies employed in previous meta-analyses regarding PCSK9 inhibitors for comparing the lipid-lowering effects of the initial three PCSK9 inhibitors with tafolecimab. Previous studies have primarily focused on comparing drug types and efficacy endpoints, overlooking the comparison of safety endpoints across different doses. In this study, our network meta-analysis encompassed multiple commonly used clinical doses of PCSK9 inhibitors to compare their therapeutic efficacy and safety, thereby addressing this research gap. Our analysis results underscored the need for further exploration into the relationship between PCSK9 inhibitor dosage and AE incidence. Furthermore, our investigation on blood lipid levels extended beyond LDL-C by incorporating ApoB and Lp(a) as evaluation indicators based on relevant research highlighting their roles as risk factors for cardiovascular disease. This comprehensive approach enhances both clinical significance and the findings’ relevance. Simultaneously, the included trials in this study have a high impact factor and citation frequency, demonstrating high reliability and evidence quality. In summary, this study provided strong evidence supporting the good lipid-lowering efficacy of PCSK9 inhibitors.

However, it was important to acknowledge the limitations of this study. Firstly, this study overlooked the fact that statin background lipid-lowering therapy is usually divided into three intensities, and didn't further analyze the impact of different intensities of statin therapy on blood lipid levels. Secondly, comparisons between different PCSK9 inhibitors and their respective doses in this study primarily relied on indirect evidence rather than direct evidence, resulting in imprecise and heterogeneous findings. Thirdly, inclisiran and tafolecimab are novel drugs with a relatively short time since market introduction, therefore there is a limited number of clinical trials with published results available for analysis in this study. Only six relevant trials were included, while several other clinical trials are still ongoing. Therefore, further updated studies are necessary to confirm the outcomes. Fourthly, regarding dosing intervals, alirocumab, evolocumab, and tafolecimab mainly adopted 2-week or 1-month cycles; whereas inclisiran had a dosing interval of up to 6 months. However, due to time constraints, this study failed to investigate its long-term therapeutic effects comprehensively. PCSK9 inhibitors and statins achieve the goal of reducing LDL-C through enhanced clearance or inhibition of synthesis, respectively. However, prolonged use of PCSK9 inhibitors alone may lead to an upregulation in LDL-C synthesis. Several related studies have also demonstrated that even when combined with statins, the decline in LDL-C exhibited a gradual attenuation over time, with an initial substantial reduction followed by a subsequent gradual picking up. Nevertheless, due to limited follow-up duration, longer-term observations are warranted to address this matter.

Our analysis findings demonstrated that ezetimibe exhibited a 25% reduction in LDL-C compared to placebo; all PCSK9 inhibitors displayed a more pronounced lipid-lowering efficacy than ezetimibe. Nevertheless, when used as an adjunctive therapy to statins, ezetimibe remained the preferred pharmacological agent. In accordance with relevant guidelines, PCSK9 inhibitors were recommended solely for extremely high-risk patients who had reached the maximum tolerable dose of ezetimibe but failed to achieve the target LDL-C level. This may be attributed to the relatively limited evidence base of PCSK9 inhibitors compared with ezetimibe, a clinically established drug with many years of usage. Furthermore, the high cost of PCSK9 inhibitors also poses an additional limitation to their clinical application. When making clinical decisions under the influence of multiple factors, although PCSK9 inhibitors have been proven to have favorable therapeutic effects in clinical practice, their high price may impede accessibility and reduce long-term patient compliance. This study only focused on the treatment strategy of PCSK9 inhibitors and can be further included in the cost-effectiveness analysis of PCSK9 inhibitors.

In recent years, with clinical evidence confirming the good efficacy of PCSK9 inhibitors in treatment, their status as statin adjuvant therapy in the guidelines has been continuously increasing. The findings of this study further support the current application of these guidelines in clinical practice and aim to provide additional evidence for making treatment decisions.

## 5 Conclusion

Compared with placebo and ezetimibe, alirocumab, evolocumab, inclisiran, and tafolecimab exhibited the ability to effectively reduce LDL-C levels in patients receiving statin background therapy, with evolocumab being the most effective treatment strategy. In terms of dosage selection, administering evolocumab at a dose of 140 mg Q2W was found to be superior to 420 mg QM. Additionally, PCSK9 inhibitors displayed significant potential in reducing ApoB and Lp(a) levels. PCSK9 inhibitors generally demonstrated favorable safety. Compared with ezetimibe, PCSK9 inhibitors exhibited similar risk characteristics regarding safety endpoints.

## Data Availability

The original contributions presented in the study are included in the article/Supplementary Material, further inquiries can be directed to the corresponding author.
