# Supplementary material for: The efficacy and safety of proprotein convertase subtilisin/kexin type 9 (PCSK9) inhibitors combined with statins in patients with hypercholesterolemia: a network meta-analysis
Source: Front Cardiovasc Med. 2024 Sep 25;11:1454918. doi: 10.3389/fcvm.2024.1454918 (PMC11461350; doi:10.3389/fcvm.2024.1454918)
Supplement: Supplementary file 1 [file Datasheet1.pdf]

Supplementary material

1 Supplementary Figures

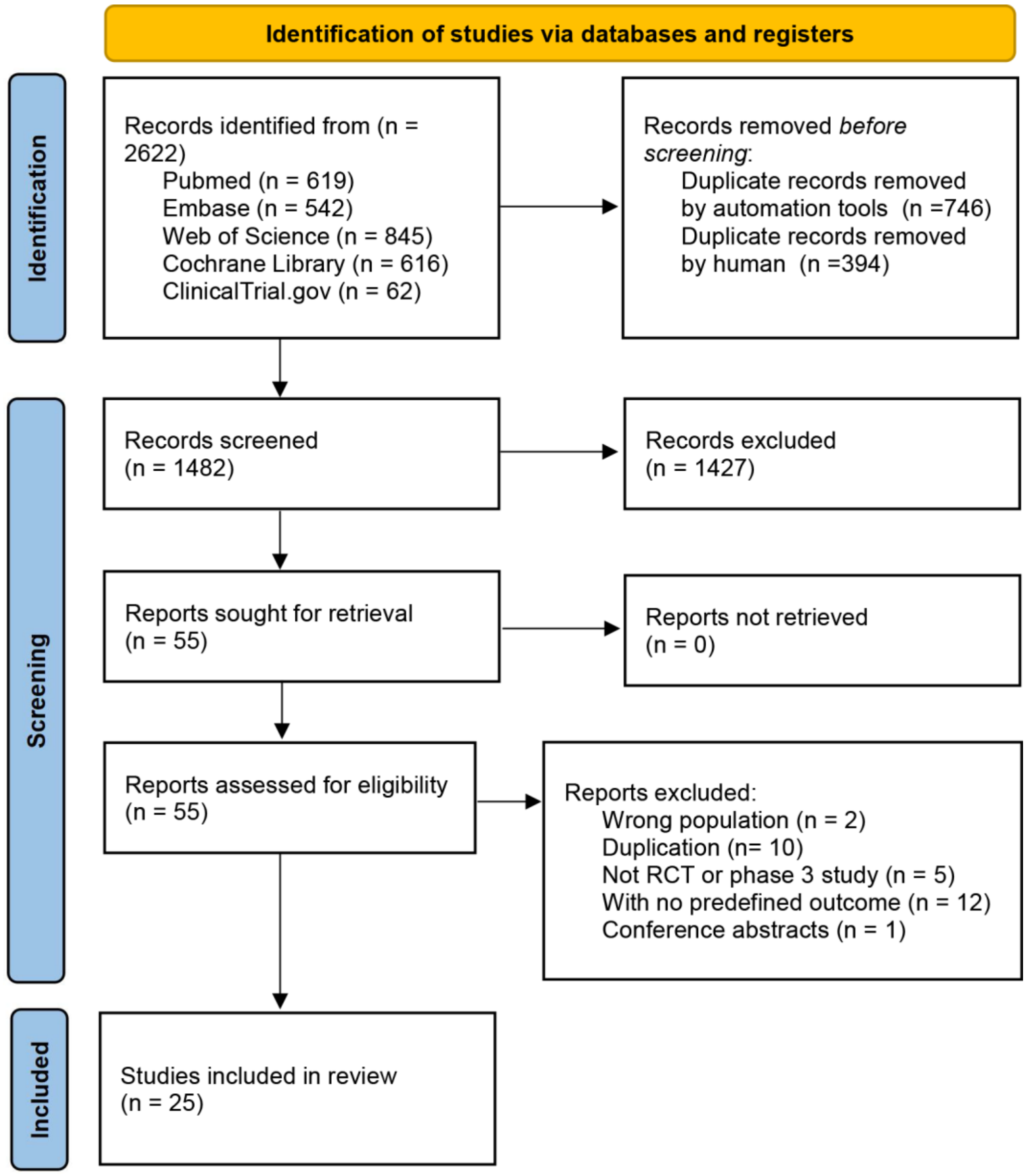

Supplementary Figure S1. PRISMA flowchart.

	Random sequence generation (selection bias)	Allocation concealment (selection bias)	Blinding of participants and personnel (performance bias)	Blinding of outcome assessment (detection bias)	Incomplete outcome data (attrition bias)	Selective reporting (reporting bias)	Other bias
BANTING	+	+	+	?	+	+	+
BERSON	+	?	+	+	+	+	+
CREDIT-1	+	?	+	+	+	+	+
CREDIT-2	+	?	+	+	+	+	+
CREDIT-4	+	?	+	+	+	+	+
DESCARTES	+	+	+	+	+	+	+
HUA TUO	+	?	+	+	+	+	+
LAPLACE-2	+	+	+	+	+	+	+
ODYSSEY CHOICE I	?	?	+	+	+	+	+
ODYSSEY COMBO I	?	?	+	+	+	+	+
ODYSSEY COMBO II	+	?	+	?	+	+	+
ODYSSEY DM-INSULIN	+	?	+	+	+	+	+
ODYSSEY EAST	+	?	+	+	+	+	+
ODYSSEY FH I,ODYSSEY FH II	+	?	+	+	+	+	+
ODYSSEY HIGH FH	+	?	+	+	+	+	+
ODYSSEY JAPAN	+	?	+	+	+	+	+
ODYSSEY KT	?	?	+	?	+	+	+
ODYSSEY LONG TERM	+	+	+	+	−	+	+
ODYSSEY OPTIONS I	+	+	+	?	+	+	+
ODYSSEY OPTIONS II	+	?	+	+	?	+	+
ORION-10,ORION-11	+	+	+	?	+	+	+
ORION-9	+	+	+	?	+	+	+
RUTHERFORD-2	+	+	+	+	+	+	+
YUKAWA-2	?	?	+	+	+	+	+

Supplementary Figure S2. Risk of bias summary for included studies.

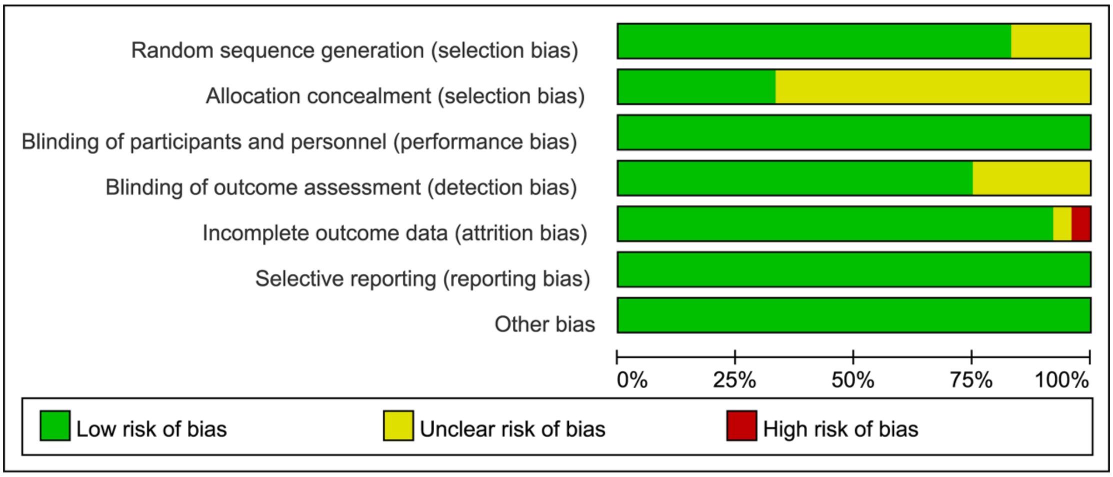

Supplementary Figure S3. Risk of bias graph of included studies.

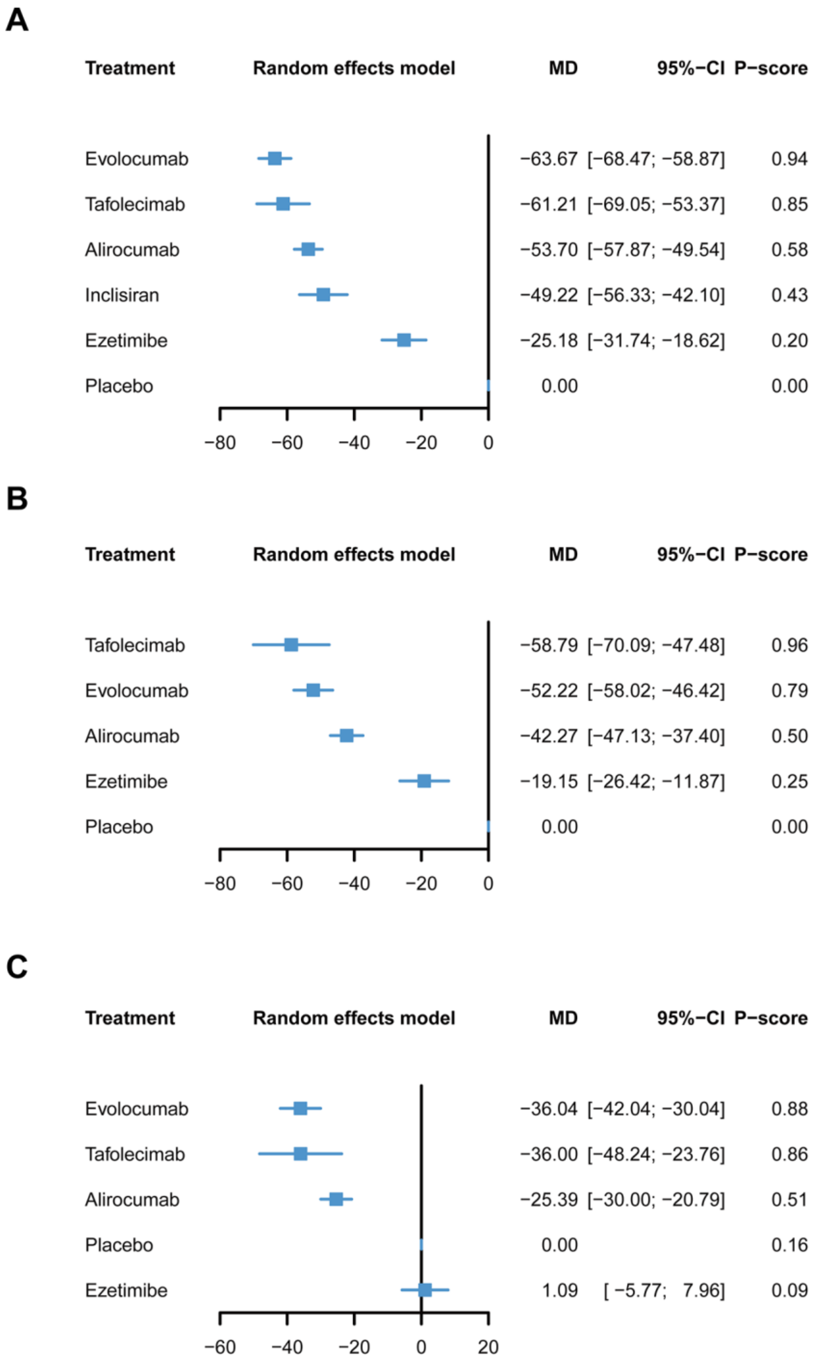

Supplementary Figure S4. Forest plot of (A) LDL-C; (B) ApoB and (C) Lp(a) in network meta-analysis.

The result is represented in mean difference (MD) and 95% confidence interval (CI) with placebo as reference.

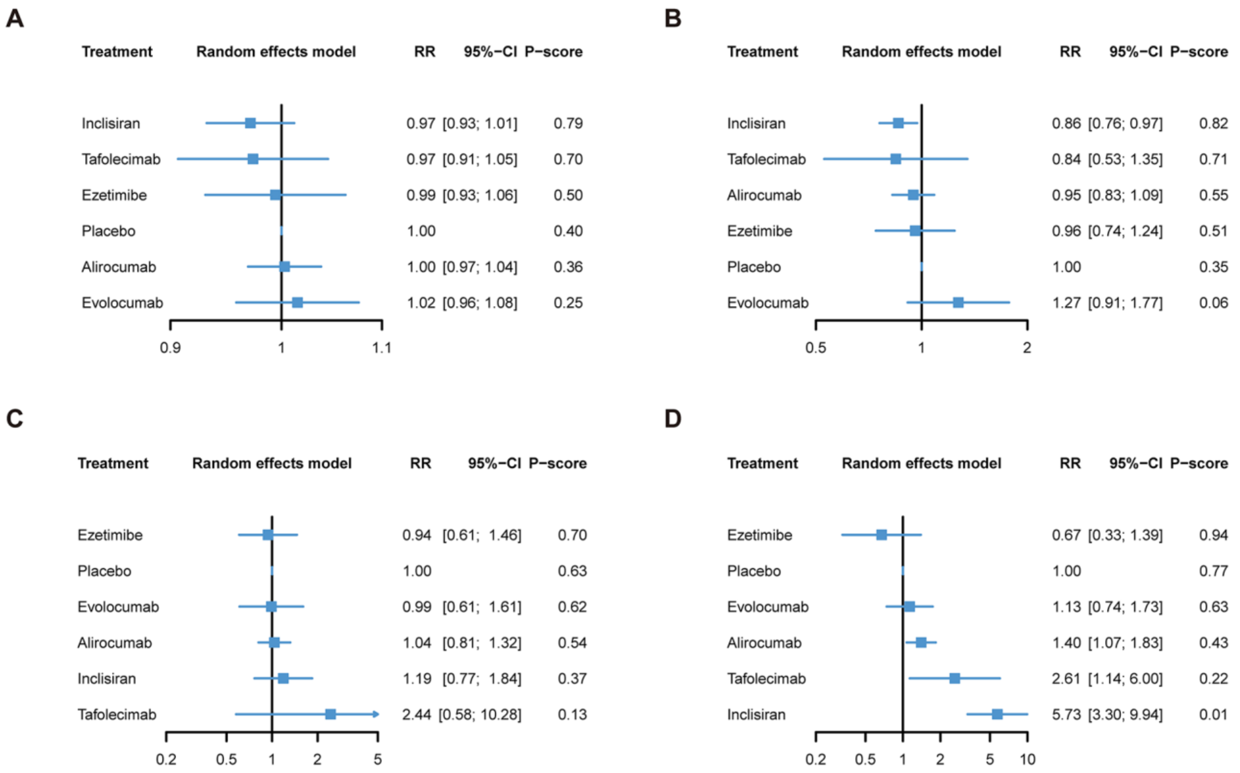

Supplementary Figure S5. Forest plot of (A) AE; (B) SAE; (C) AE leading to treatment discontinuation and (D) intection-site reaction. The result is represented in risk ratio (RR) and 95% confidence interval (CI) with placebo as reference.

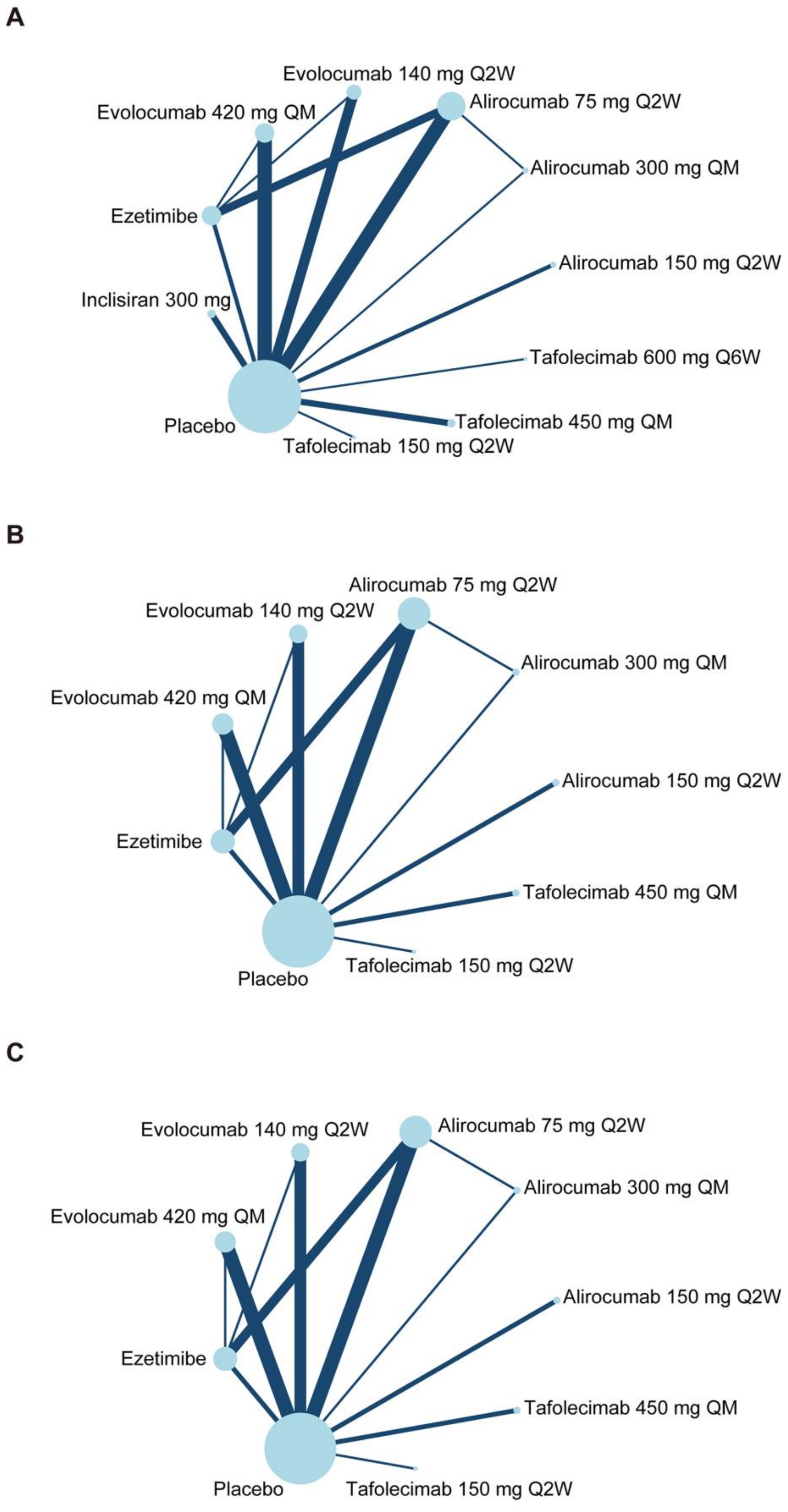

Supplementary Figure S6. Network geometry of (A) LDL-C; (B) ApoB and (C) Lp(a) (different drug dose).

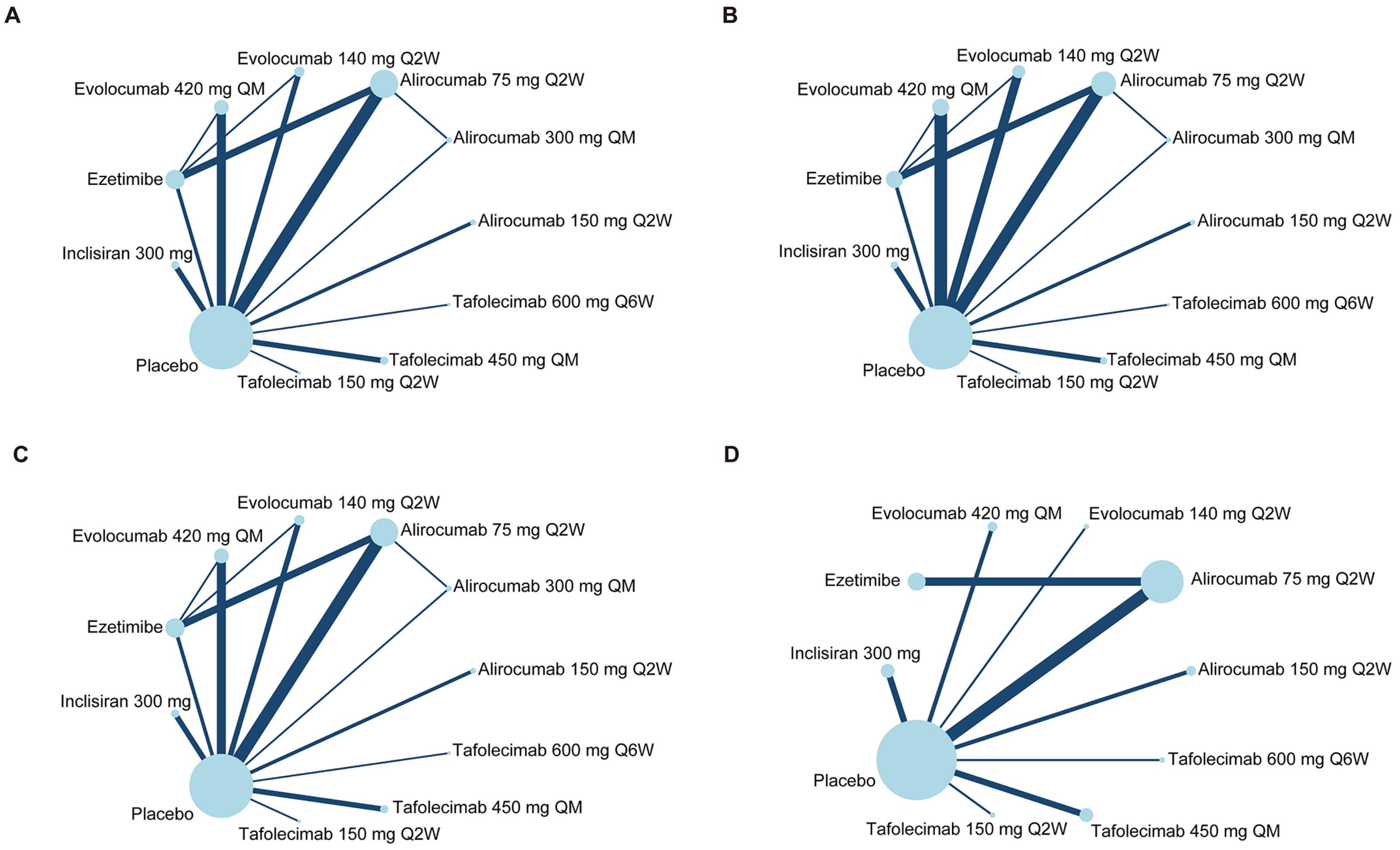

Supplementary Figure S7. Network geometry of (A) AE; (B) SAE; (C) AE leading to treatment discontinuation and (D) intection-site reaction (different drug dose).

A

Alirocumab 75 mg Q2W										
4.24 (-5.28; 13.75)	Alirocumab 150 mg Q2W									
6.61 (-3.68; 16.90)	2.37 (-10.99; 15.73)	Alirocumab 300 mg QM								
**16.64 (10.10; 23.18)**	**12.40 (2.22; 22.59)**	10.03 (-1.46; 21.53)	Evolocumab 140 mg Q2W							
**9.02 (3.18; 14.86)**	4.78 (-4.93; 14.49)	2.41 (-8.68; 13.51)	**-7.62 (-14.58; -0.66)**	Evolocumab 420 mg QM						
-3.26 (-10.63; 4.12)	-7.49 (-18.10; 3.12)	-9.86 (-21.79; 2.07)	**-19.90 (-28.12; -11.68)**	**-12.28 (-19.90; -4.65)**	Inclisiran 300 mg					
4.91 (-12.71; 22.53)	0.67 (-18.53; 19.88)	-1.70 (-21.66; 18.26)	-11.73 (-29.73; 6.26)	-4.11 (-21.84; 13.62)	8.16 (-10.07; 26.40)	Tafolecimab 150 mg Q2W				
**11.44 (3.00; 19.89)**	7.21 (-4.17; 18.59)	4.84 (-7.78; 17.45)	-5.20 (-14.39; 3.99)	2.42 (-6.24; 11.09)	**14.70 (5.04; 24.36)**	6.54 (-12.16; 25.23)	Tafolecimab 450 mg QM			
2.21 (-10.69; 15.10)	-2.03 (-17.01; 12.96)	-4.40 (-20.34; 11.55)	**-14.43 (-27.83; -1.04)**	-6.81 (-19.85; 6.23)	5.46 (-8.26; 19.19)	-2.70 (-23.78; 18.38)	-9.24 (-23.56; 5.09)	Tafolecimab 600 mg Q6W		
**-28.02 (-32.98; -23.06)**	**-32.26 (-42.38; -22.13)**	**-34.63 (-45.74; -23.52)**	**-44.66 (-51.70; -37.62)**	**-37.04 (-43.55; -30.52)**	**-24.76 (-32.90; -16.62)**	**-32.93 (-50.88; -14.97)**	**-39.46 (-48.58; -30.34)**	**-30.23 (-43.58; -16.88)**	Ezetimibe	
**-52.49 (-56.50; -48.48)**	**-56.73 (-65.35; -48.10)**	**-59.10 (-69.30; -48.90)**	**-69.13 (-74.55; -63.72)**	**-61.51 (-65.97; -57.05)**	**-49.24 (-55.42; -43.05)**	**-57.40 (-74.56; -40.24)**	**-63.94 (-71.36; -56.51)**	**-54.70 (-66.95; -42.45)**	**-24.47 (-29.77; -19.18)**	Placebo

B

Alirocumab 75 mg Q2W								
5.34 (-4.47; 15.15)	Alirocumab 150 mg Q2W							
6.01 (-4.55; 16.57)	0.67 (-13.01; 14.35)	Alirocumab 300 mg QM						
**15.40 (8.60; 22.19)**	10.05 (-0.31; 20.42)	9.39 (-2.40; 21.17)	Evolocumab 140 mg Q2W					
**9.57 (3.15; 15.99)**	4.23 (-5.87; 14.32)	3.56 (-8.00; 15.12)	-5.83 (-13.19; 1.54)	Evolocumab 420 mg QM				
21.03 (-2.07; 44.12)	15.68 (-8.64; 40.00)	15.01 (-9.98; 40.00)	5.63 (-17.71; 28.97)	11.46 (-11.76; 34.68)	Tafolecimab 150 mg Q2W			
**17.37 (6.56; 28.19)**	12.03 (-1.20; 25.26)	11.36 (-3.06; 25.78)	1.98 (-9.35; 13.30)	7.81 (-3.27; 18.88)	-3.65 (-28.39; 21.09)	Tafolecimab 450 mg QM		
**-22.73 (-27.71; -17.75)**	**-28.07 (-38.39; -17.75)**	**-28.74 (-40.08; -17.41)**	**-38.13 (-45.30; -30.95)**	**-32.30 (-39.19; -25.41)**	**-43.76 (-67.07; -20.44)**	**-40.10 (-51.39; -28.82)**	Ezetimibe	
**-40.67 (-45.05; -36.30)**	**-46.02 (-54.80; -37.24)**	**-46.69 (-57.18; -36.20)**	**-56.07 (-61.58; -50.56)**	**-50.24 (-55.23; -45.26)**	**-61.70 (-84.38; -39.02)**	**-58.05 (-67.94; -48.16)**	**-17.94 (-23.37; -12.52)**	Placebo

C

Alirocumab 75 mg Q2W								
-1.34 (-13.87; 11.19)	Alirocumab 150 mg Q2W							
4.09 (-9.27; 17.44)	5.43 (-11.95; 22.81)	Alirocumab 300 mg QM						
**13.36 (4.05; 22.68)**	**14.71 (0.97; 28.44)**	9.28 (-6.07; 24.63)	Evolocumab 140 mg Q2W					
**8.68 (0.09; 17.27)**	10.02 (-3.17; 23.21)	4.59 (-10.29; 19.48)	-4.69 (-15.09; 5.72)	Evolocumab 420 mg QM				
18.11 (-8.00; 44.22)	19.45 (-8.42; 47.32)	14.02 (-14.73; 42.78)	4.74 (-21.97; 31.46)	9.43 (-17.01; 35.87)	Tafolecimab 150 mg Q2W			
8.77 (-6.41; 23.95)	10.11 (-7.93; 28.15)	4.68 (-14.70; 24.06)	-4.59 (-20.79; 11.60)	0.09 (-15.64; 15.82)	-9.34 (-38.50; 19.82)	Tafolecimab 450 mg QM		
**-26.15 (-32.52; -19.78)**	**-24.81 (-37.99; -11.63)**	**-30.24 (-44.59; -15.88)**	**-39.51 (-49.15; -29.88)**	**-34.83 (-43.91; -25.75)**	**-44.26 (-70.69; -17.83)**	**-34.92 (-50.64; -19.20)**	Ezetimibe	
**-25.19 (-30.76; -19.62)**	**-23.85 (-35.08; -12.63)**	**-29.28 (-42.55; -16.01)**	**-38.56 (-46.48; -30.64)**	**-33.87 (-40.80; -26.94)**	**-43.30 (-68.81; -17.79)**	**-33.96 (-48.09; -19.84)**	0.96 (-5.95; 7.86)	Placebo

Supplementary Figure S8. League table of (A) LDL-C; (B) ApoB and (C) Lp(a) (different drug dose).

The result is represented in mean difference (MD) and 95% confidence interval (CI). Significant pairwise comparisons are highlighted.

A

Alirocumab 75 mg Q2W										
1.04 (0.97; 1.10)	Alirocumab 150 mg Q2W									
0.92 (0.83; 1.02)	**0.89 (0.80; 0.99)**	Alirocumab 300 mg QM								
1.04 (0.90; 1.19)	1.00 (0.87; 1.15)	1.12 (0.95; 1.33)	Evolocumab 140 mg Q2W							
1.00 (0.92; 1.09)	0.97 (0.89; 1.05)	1.09 (0.96; 1.22)	0.97 (0.83; 1.13)	Evolocumab 420 mg QM						
1.05 (0.99; 1.11)	1.01 (0.96; 1.07)	**1.14 (1.02; 1.26)**	1.01 (0.88; 1.17)	1.05 (0.97; 1.13)	Inclisiran 300 mg					
0.83 (0.55; 1.24)	0.80 (0.53; 1.20)	0.90 (0.59; 1.36)	0.80 (0.52; 1.22)	0.83 (0.55; 1.24)	0.79 (0.53; 1.18)	Tafolecimab 150 mg Q2W				
**1.11 (1.01; 1.22)**	1.07 (0.98; 1.17)	**1.20 (1.06; 1.36)**	1.07 (0.91; 1.26)	1.11 (0.99; 1.23)	1.06 (0.97; 1.15)	1.34 (0.89; 2.02)	Tafolecimab 450 mg QM			
0.96 (0.86; 1.09)	0.93 (0.83; 1.04)	1.04 (0.90; 1.21)	0.93 (0.78; 1.11)	0.96 (0.84; 1.09)	0.92 (0.82; 1.03)	1.16 (0.77; 1.76)	**0.87 (0.76; 0.99)**	Tafolecimab 600 mg Q6W		
1.01 (0.96; 1.07)	0.97 (0.90; 1.06)	1.09 (0.97; 1.23)	0.97 (0.84; 1.13)	1.01 (0.91; 1.11)	0.96 (0.89; 1.04)	1.22 (0.81; 1.83)	0.91 (0.82; 1.01)	1.05 (0.92; 1.19)	Ezetimibe	
1.01 (0.97; 1.06)	0.98 (0.94; 1.02)	1.10 (1.00; 1.21)	0.98 (0.85; 1.12)	1.01 (0.94; 1.09)	0.97 (0.93; 1.00)	1.22 (0.82; 1.83)	**0.91 (0.84; 0.99)**	1.05 (0.94; 1.17)	1.00 (0.94; 1.08)	Placebo

B

Alirocumab 75 mg Q2W										
0.98 (0.69; 1.38)	Alirocumab 150 mg Q2W									
1.21 (0.72; 2.01)	1.24 (0.72; 2.12)	Alirocumab 300 mg QM								
0.95 (0.51; 1.77)	0.97 (0.52; 1.82)	0.79 (0.37; 1.67)	Evolocumab 140 mg Q2W							
0.76 (0.46; 1.24)	0.78 (0.48; 1.27)	0.63 (0.33; 1.20)	0.80 (0.39; 1.64)	Evolocumab 420 mg QM						
1.14 (0.84; 1.54)	1.16 (0.87; 1.56)	0.94 (0.57; 1.57)	1.20 (0.66; 2.19)	1.50 (0.95; 2.37)	Inclisiran 300 mg					
0.42 (0.02; 8.60)	0.43 (0.02; 8.79)	0.35 (0.02; 7.33)	0.44 (0.02; 9.46)	0.56 (0.03; 11.56)	0.37 (0.02; 7.52)	Tafolecimab 150 mg Q2W				
1.47 (0.75; 2.90)	1.51 (0.77; 2.96)	1.22 (0.55; 2.70)	1.55 (0.66; 3.64)	1.94 (0.91; 4.15)	1.29 (0.67; 2.48)	3.48 (0.16; 74.81)	Tafolecimab 450 mg QM			
0.80 (0.36; 1.79)	0.82 (0.37; 1.83)	0.67 (0.27; 1.64)	0.85 (0.32; 2.20)	1.06 (0.44; 2.54)	0.71 (0.32; 1.54)	1.90 (0.09; 42.07)	0.55 (0.20; 1.47)	Tafolecimab 600 mg Q6W		
0.98 (0.76; 1.26)	1.00 (0.66; 1.53)	0.81 (0.46; 1.43)	1.03 (0.53; 2.01)	1.29 (0.75; 2.23)	0.86 (0.58; 1.27)	2.32 (0.11; 47.64)	0.67 (0.32; 1.37)	1.22 (0.53; 2.83)	Ezetimibe	
0.95 (0.74; 1.21)	0.97 (0.76; 1.23)	0.78 (0.48; 1.27)	1.00 (0.56; 1.78)	1.25 (0.81; 1.91)	**0.83 (0.70; 0.99)**	2.24 (0.11; 45.05)	0.64 (0.34; 1.21)	1.18 (0.55; 2.53)	0.96 (0.68; 1.37)	Placebo

C

Alirocumab 75 mg Q2W										
0.77 (0.46; 1.28)	Alirocumab 150 mg Q2W									
1.28 (0.62; 2.63)	1.67 (0.80; 3.48)	Alirocumab 300 mg QM								
0.96 (0.39; 2.34)	1.25 (0.50; 3.12)	0.75 (0.26; 2.18)	Evolocumab 140 mg Q2W							
0.84 (0.36; 1.95)	1.09 (0.48; 2.51)	0.65 (0.24; 1.78)	0.88 (0.28; 2.74)	Evolocumab 420 mg QM						
0.78 (0.43; 1.40)	1.01 (0.59; 1.75)	0.61 (0.28; 1.34)	0.81 (0.31; 2.13)	0.93 (0.39; 2.24)	Inclisiran 300 mg					
0.41 (0.02; 8.48)	0.54 (0.03; 10.97)	0.32 (0.01; 6.93)	0.43 (0.02; 9.75)	0.49 (0.02; 10.86)	0.53 (0.03; 10.97)	Tafolecimab 150 mg Q2W				
0.55 (0.10; 3.06)	0.72 (0.13; 3.94)	0.43 (0.07; 2.59)	0.58 (0.09; 3.77)	0.66 (0.11; 4.13)	0.71 (0.13; 3.98)	1.34 (0.04; 41.40)	Tafolecimab 450 mg QM			
0.17 (0.01; 3.05)	0.22 (0.01; 3.95)	0.13 (0.01; 2.50)	0.17 (0.01; 3.52)	0.20 (0.01; 3.92)	0.21 (0.01; 3.95)	0.40 (0.01; 25.76)	0.30 (0.01; 8.35)	Tafolecimab 600 mg Q6W		
1.08 (0.73; 1.59)	1.41 (0.76; 2.61)	0.84 (0.38; 1.87)	1.13 (0.45; 2.80)	1.29 (0.52; 3.17)	1.39 (0.70; 2.75)	2.61 (0.12; 54.76)	1.94 (0.34; 11.10)	6.50 (0.35; 122.03)	Ezetimibe	
0.92 (0.62; 1.37)	1.20 (0.87; 1.67)	0.72 (0.37; 1.39)	0.97 (0.41; 2.28)	1.10 (0.51; 2.37)	1.19 (0.77; 1.84)	2.24 (0.11; 44.83)	1.66 (0.32; 8.77)	5.57 (0.31; 99.71)	0.86 (0.51; 1.45)	Placebo

D

Alirocumab 75 mg Q2W									
0.99 (0.57; 1.71)	Alirocumab 150 mg Q2W								
1.13 (0.22; 5.94)	1.14 (0.22; 5.97)	Evolocumab 140 mg Q2W							
1.10 (0.54; 2.23)	1.11 (0.56; 2.23)	0.97 (0.18; 5.38)	Evolocumab 420 mg QM						
**0.24 (0.12; 0.48)**	**0.25 (0.13; 0.48)**	0.21 (0.04; 1.17)	**0.22 (0.10; 0.49)**	Inclisiran 300 mg					
0.62 (0.03; 12.78)	0.63 (0.03; 12.88)	0.55 (0.02; 16.45)	0.56 (0.03; 11.95)	2.56 (0.12; 53.94)	Tafolecimab 150 mg Q2W				
0.65 (0.19; 2.21)	0.65 (0.19; 2.21)	0.57 (0.08; 4.14)	0.59 (0.16; 2.15)	2.66 (0.73; 9.63)	1.04 (0.04; 25.84)	Tafolecimab 450 mg QM			
0.49 (0.14; 1.73)	0.49 (0.14; 1.73)	0.43 (0.06; 3.20)	0.44 (0.12; 1.68)	2.00 (0.53; 7.53)	0.78 (0.03; 19.77)	0.75 (0.14; 4.02)	Tafolecimab 600 mg Q6W		
2.11 (0.96; 4.65)	2.13 (0.82; 5.56)	1.86 (0.30; 11.65)	1.92 (0.67; 5.52)	**8.70 (3.08; 24.60)**	3.40 (0.15; 77.26)	3.28 (0.76; 14.10)	4.34 (0.98; 19.32)	Ezetimibe	
1.39 (0.94; 2.07)	1.41 (0.96; 2.05)	1.23 (0.25; 6.12)	1.26 (0.70; 2.27)	**5.73 (3.30; 9.94)**	2.24 (0.11; 44.83)	2.16 (0.67; 6.91)	2.86 (0.86; 9.54)	0.66 (0.27; 1.59)	Placebo

Supplementary Figure S9. League table of (A) AE; (B) SAE; (C) AE leading to treatment discontinuation and (D) injection-site reaction (different drug dose).

The result is represented in risk ratio (RR) and 95% confidence interval (CI). Significant pairwise comparisons are highlighted.

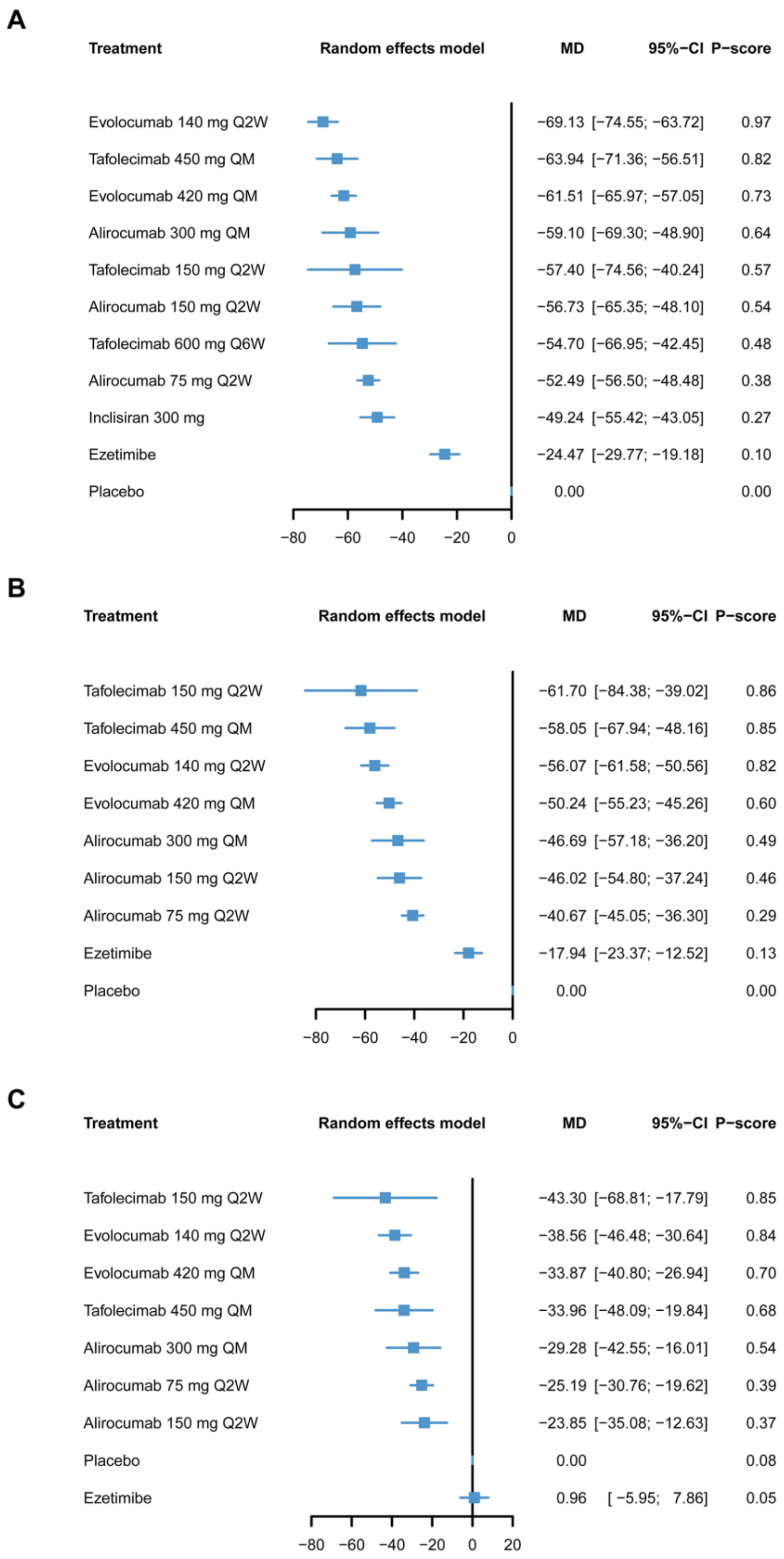

Supplementary Figure S10. Forest plot of (A) LDL-C; (B) ApoB and (C) Lp(a) in network meta-analysis (different drug dose).

The result is represented in mean difference (MD) and 95% confidence interval (CI) with placebo as reference.

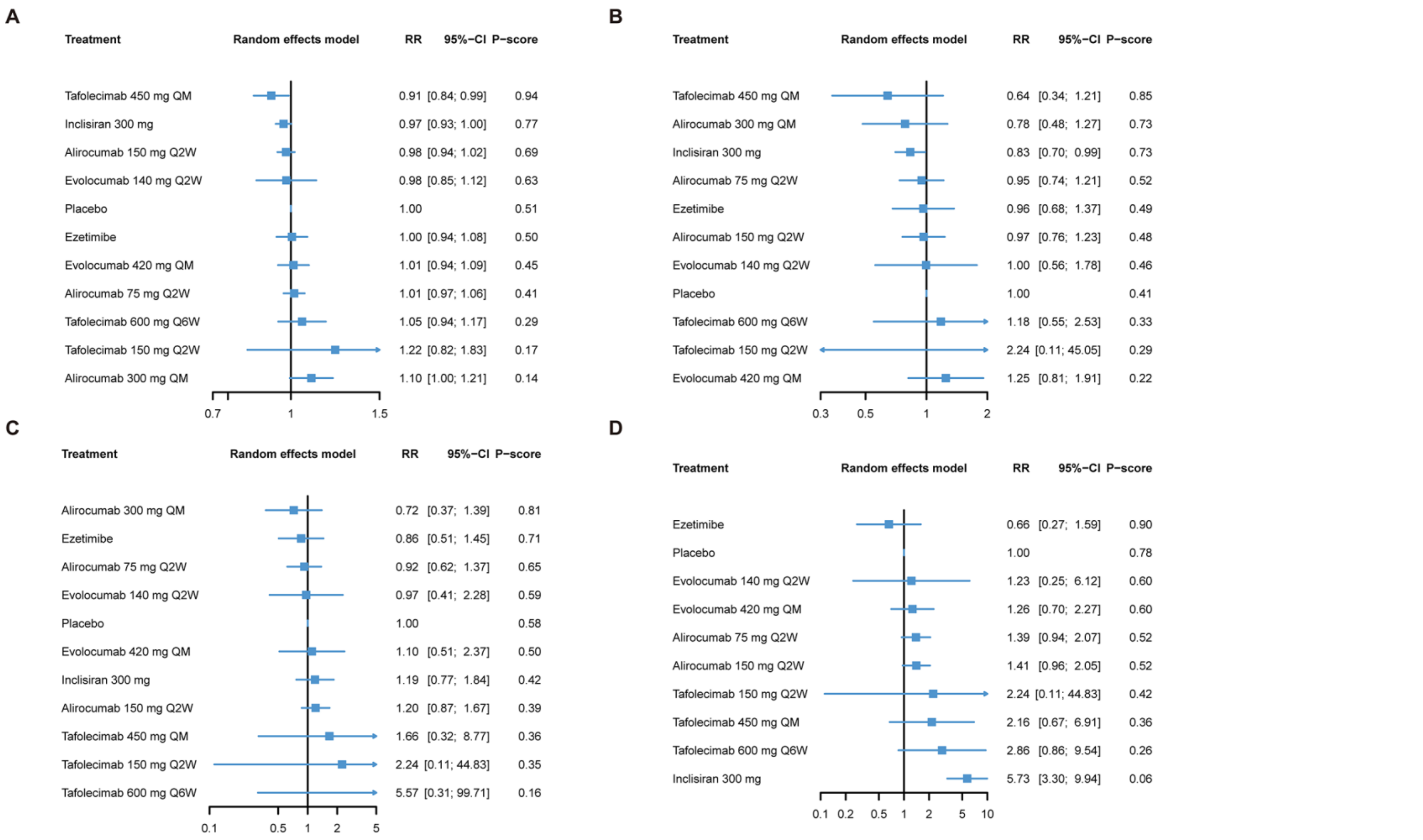

Supplementary Figure S11. Forest plot of (A) AE; (B) SAE; (C) AE leading to treatment discontinuation and (D) injection-site reaction (different drug dose).

The result is represented in risk ratio (RR) and 95% confidence interval (CI) with placebo as reference.

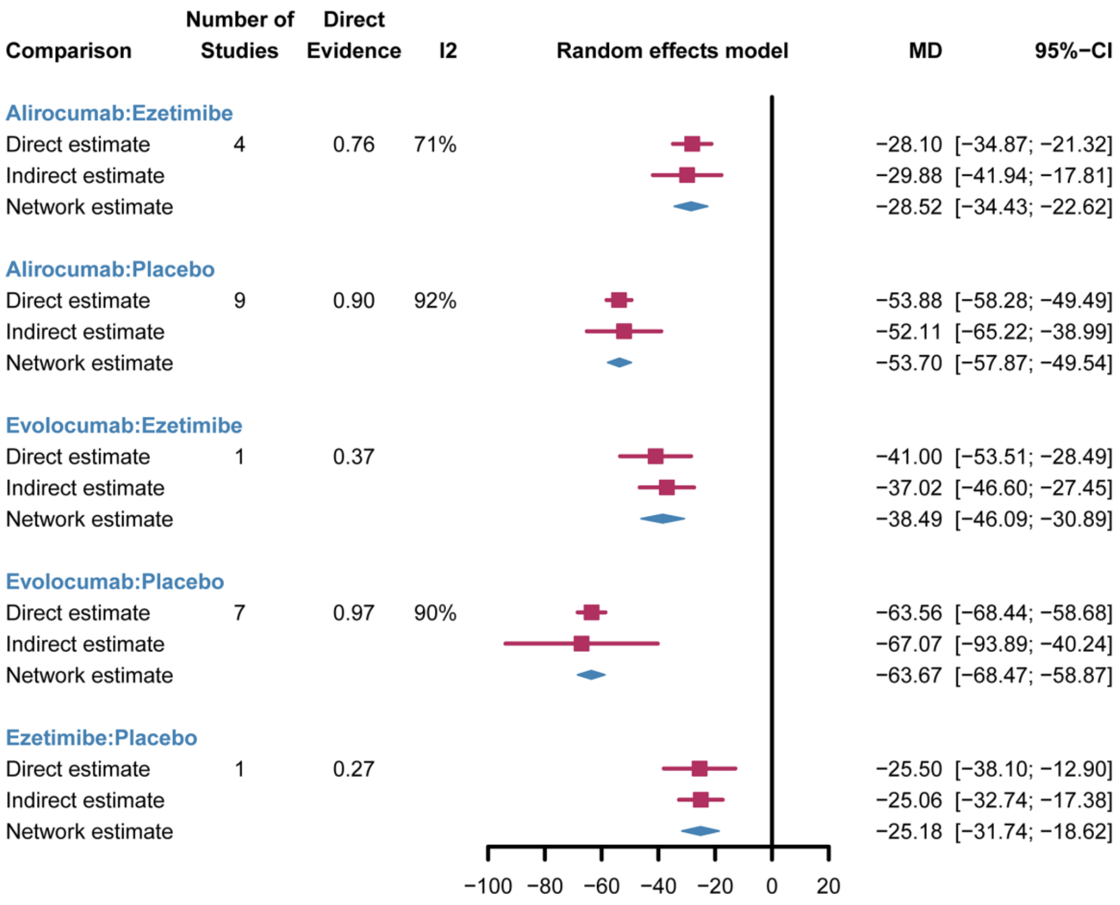

Supplementary Figure S12. Direct and indirect evidence for estimating the percentage change in LDL-C.

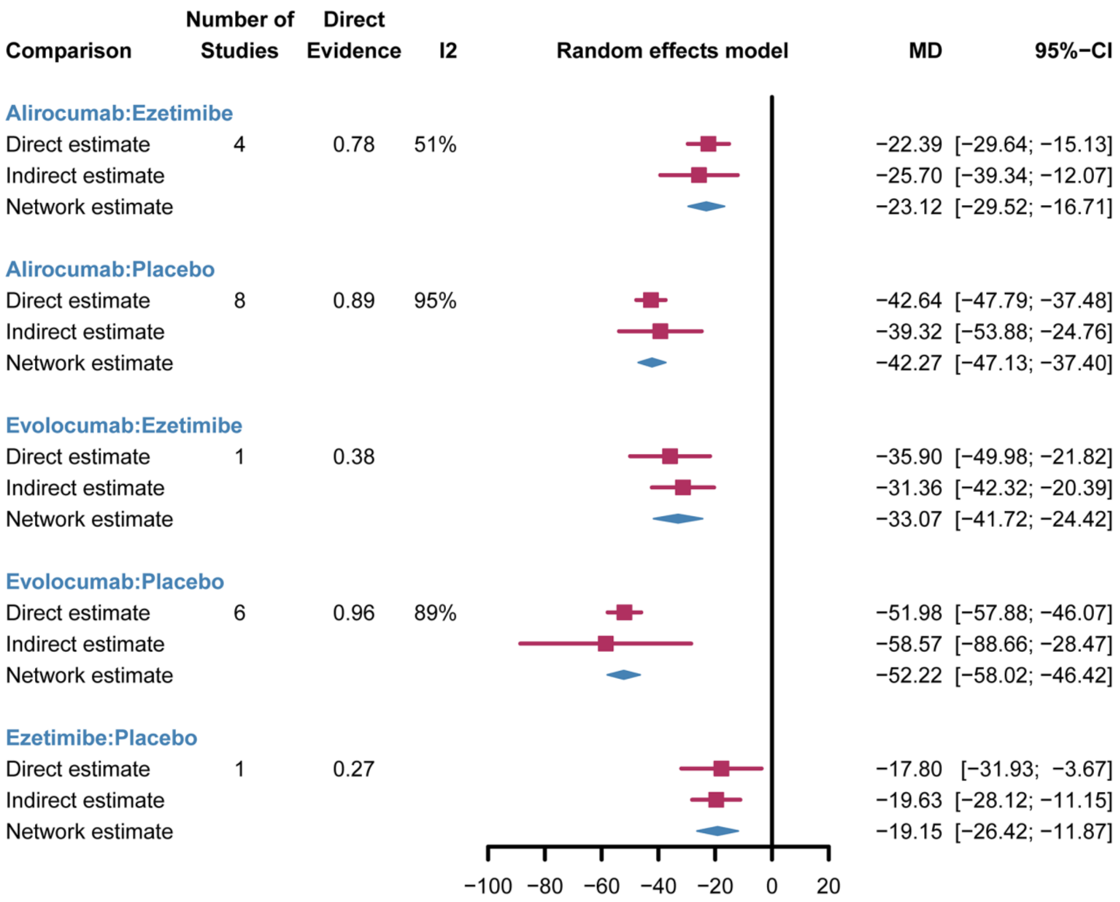

Supplementary Figure S13. Direct and indirect evidence for estimating the percentage change in ApoB.

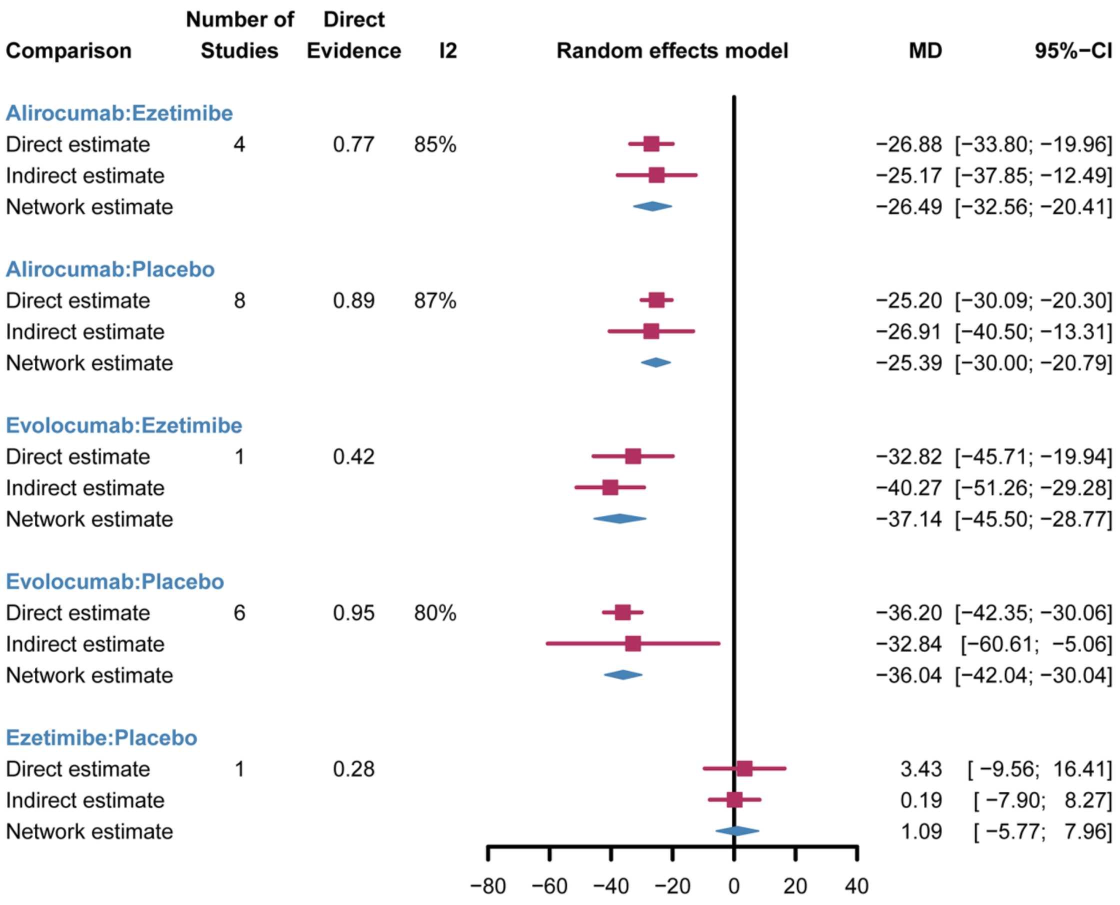

Supplementary Figure S14. Direct and indirect evidence for estimating the percentage change in Lp(a).

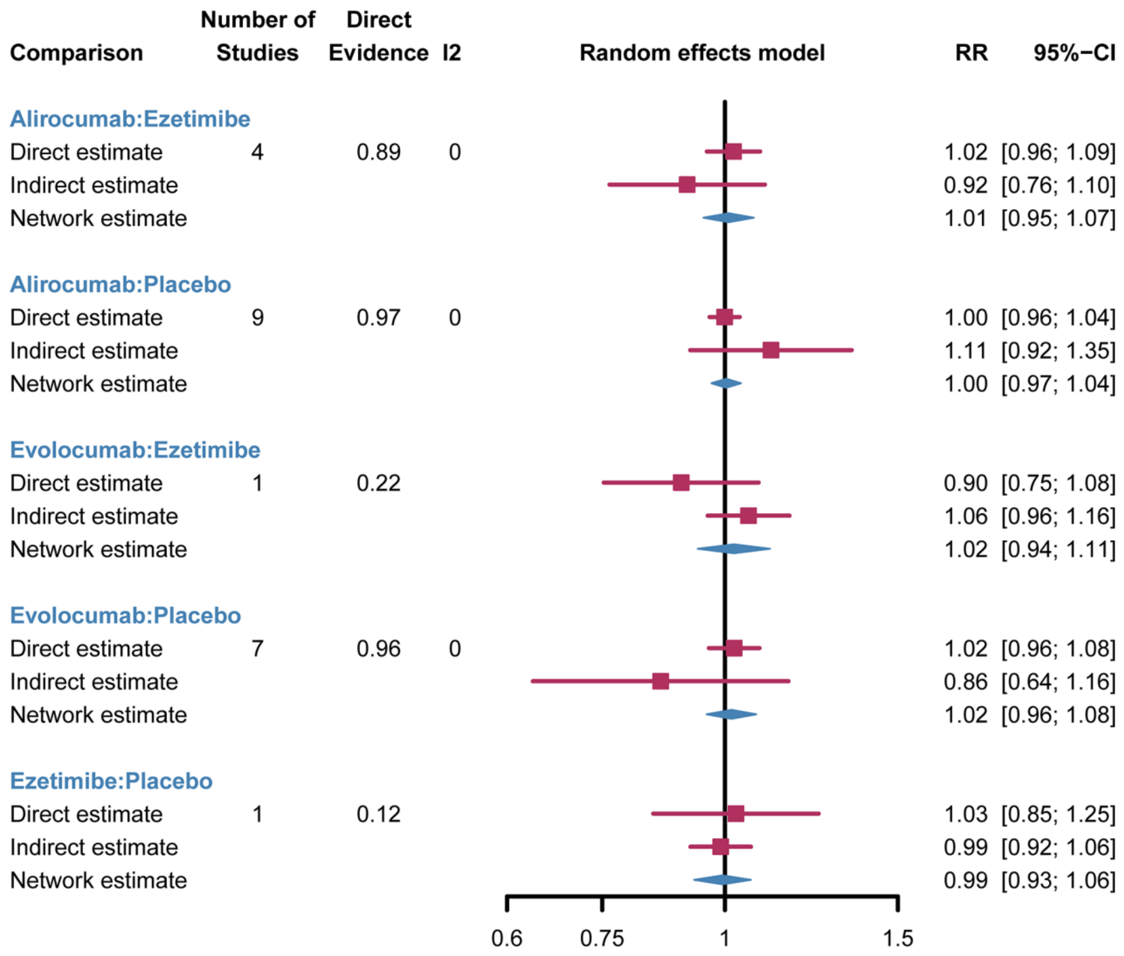

Supplementary Figure S15. Direct and indirect evidence for estimating the risk ratio of AE.

Comparison	Number of Studies	Direct Evidence	I2	RR	95%−CI
**Alirocumab:Ezetimibe**					
Direct estimate	4	0.98	0%	0.97	[0.78; 1.21]
Indirect estimate				1.97	[0.47; 8.33]
Network estimate				0.99	[0.79; 1.23]
**Alirocumab:Placebo**					
Direct estimate	9	0.99	0%	0.95	[0.83; 1.09]
Indirect estimate				0.47	[0.11; 2.00]
Network estimate				0.95	[0.83; 1.09]
**Evolocumab:Ezetimibe**					
Direct estimate	1	0.08		2.28	[0.54; 9.58]
Indirect estimate				1.26	[0.82; 1.95]
Network estimate				1.33	[0.88; 2.01]
**Evolocumab:Placebo**					
Direct estimate	7	0.98	23%	1.24	[0.89; 1.74]
Indirect estimate				3.77	[0.39; 36.07]
Network estimate				1.27	[0.91; 1.77]
**Ezetimibe:Placebo**					
Direct estimate	1	0.03		0.39	[0.09; 1.71]
Indirect estimate				0.99	[0.76; 1.28]
Network estimate				0.96	[0.74; 1.24]

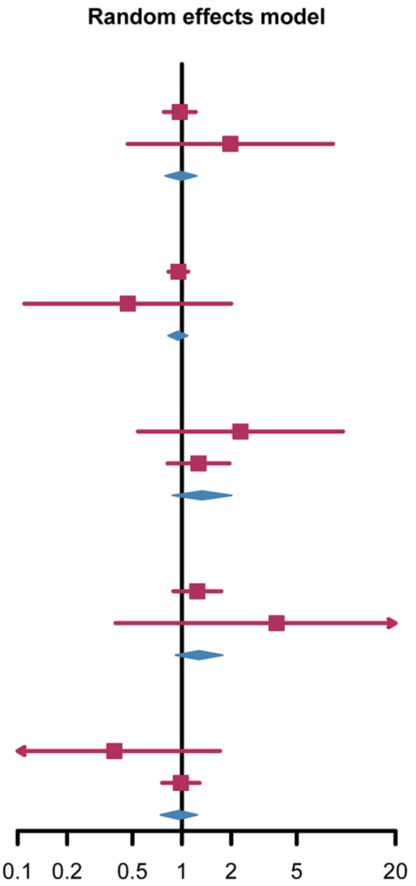

Supplementary Figure S16. Direct and indirect evidence for estimating the risk ratio of SAE.

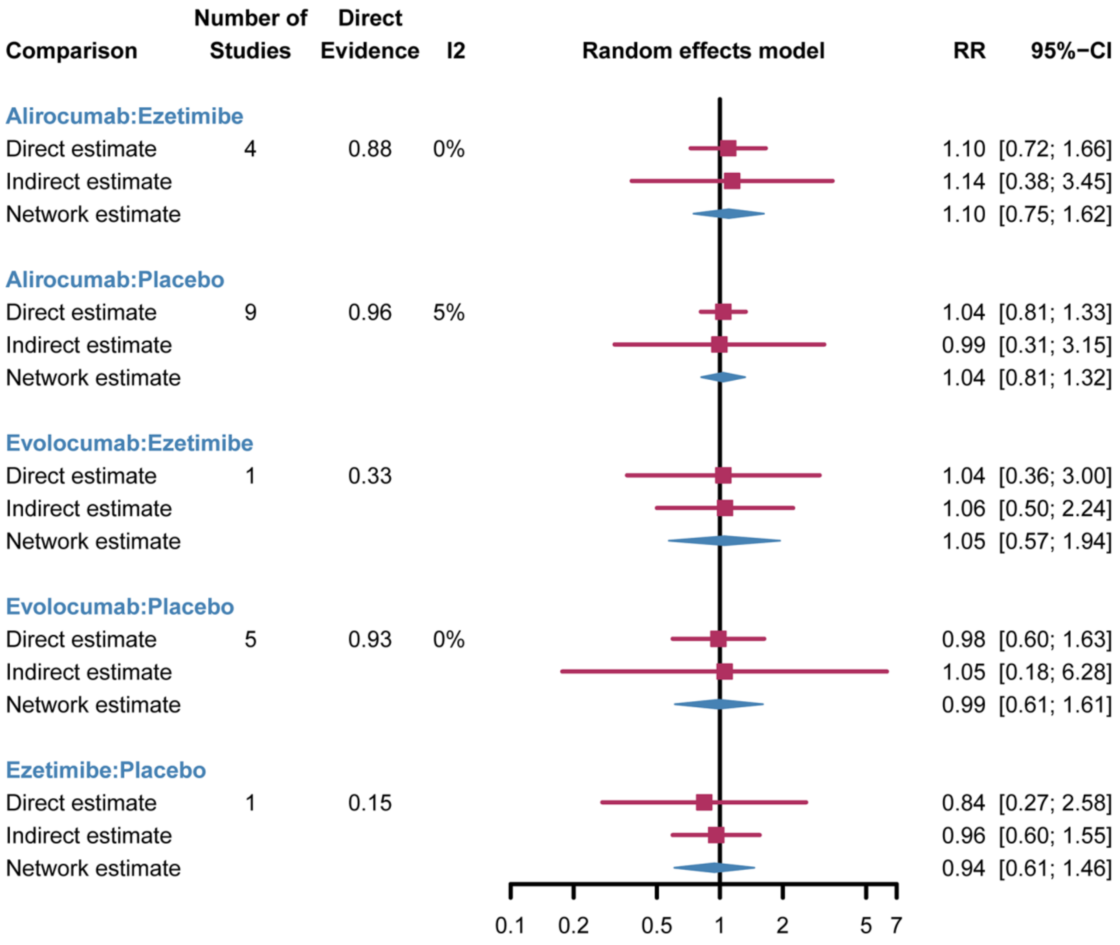

Supplementary Figure S17. Direct and indirect evidence for estimating the risk ratio of AE leading to treatment discontinuation.

Comparison	Number of Studies	Direct Evidence	I2	RR	95%−CI
**Alirocumab:Ezetimibe**					
Direct estimate	4	0.78	0	2.11	[0.96; 4.65]
Indirect estimate				1.95	[0.44; 8.67]
Network estimate				2.08	[1.04; 4.17]
**Alirocumab:Placebo**					
Direct estimate	7	0.97	0	1.40	[1.07; 1.84]
Indirect estimate				1.51	[0.29; 7.99]
Network estimate				1.40	[1.07; 1.83]
**Evolocumab:Ezetimibe**					
Direct estimate	1	0.30		1.48	[0.34; 6.44]
Indirect estimate				1.77	[0.68; 4.63]
Network estimate				1.68	[0.75; 3.75]
**Evolocumab:Placebo**					
Direct estimate	4	0.97	0	1.14	[0.74; 1.75]
Indirect estimate				1.01	[0.08; 12.90]
Network estimate				1.13	[0.74; 1.73]
**Ezetimibe:Placebo**					
Direct estimate	1	0.22		0.63	[0.14; 2.95]
Indirect estimate				0.69	[0.30; 1.56]
Network estimate				0.67	[0.33; 1.39]

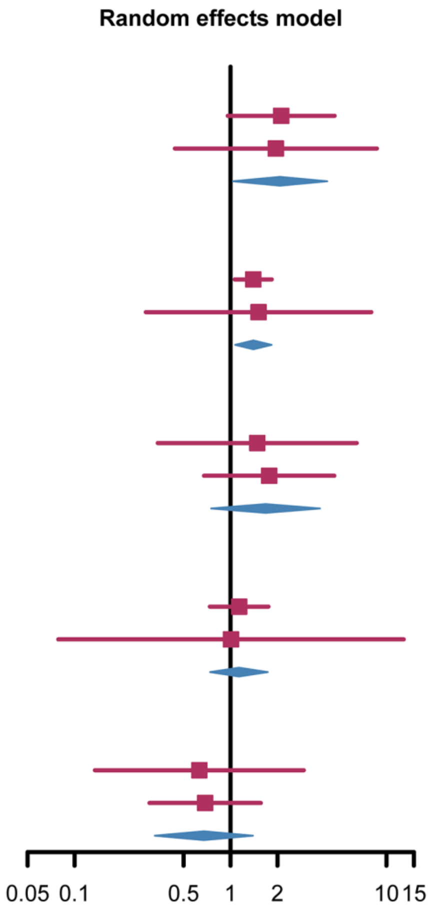

Supplementary Figure S18. Direct and indirect evidence for estimating the risk ratio of injection-site reaction.

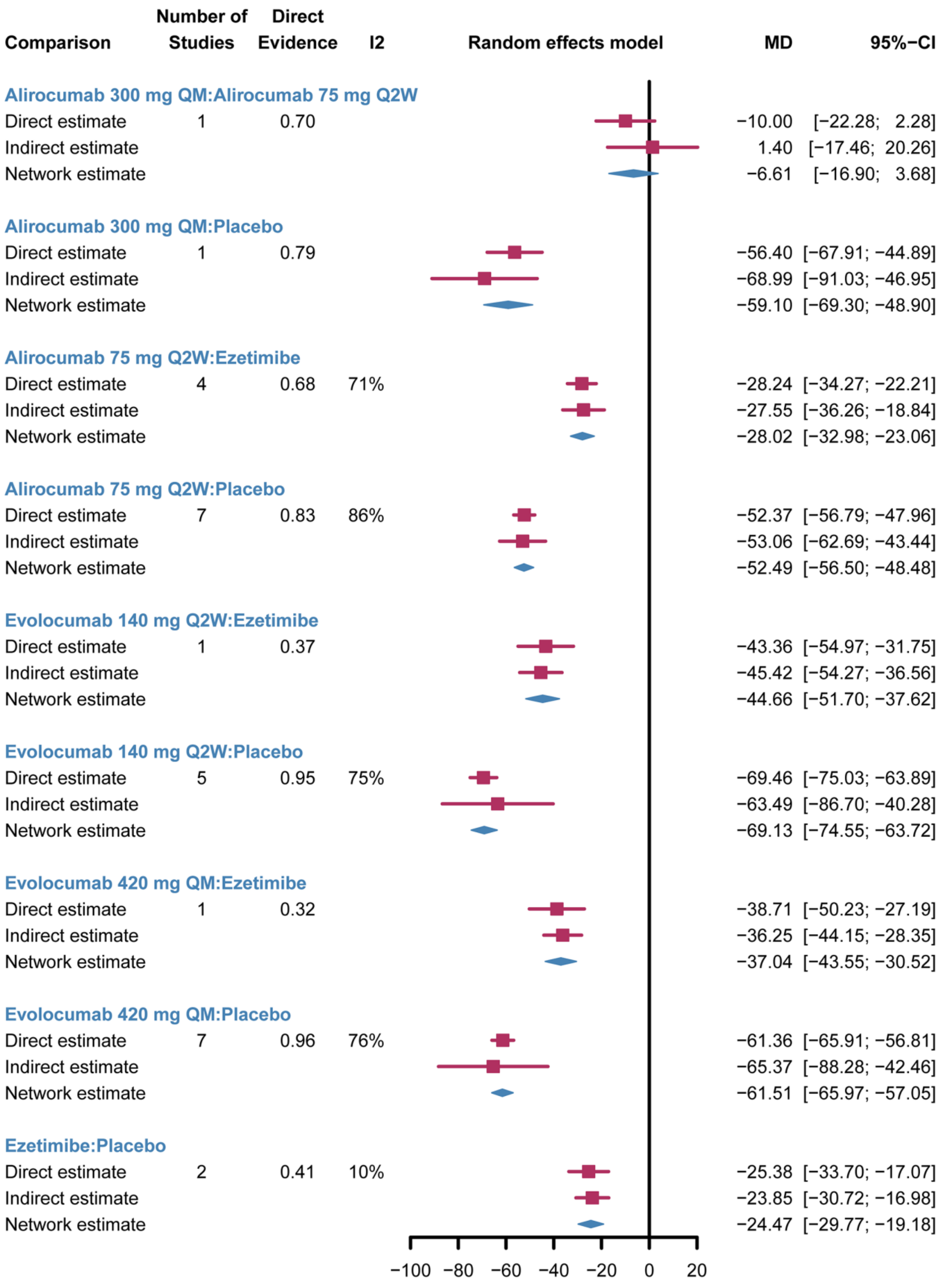

Supplementary Figure S19. Direct and indirect evidence for estimating the percentage change in LDL-C (different drug dose).

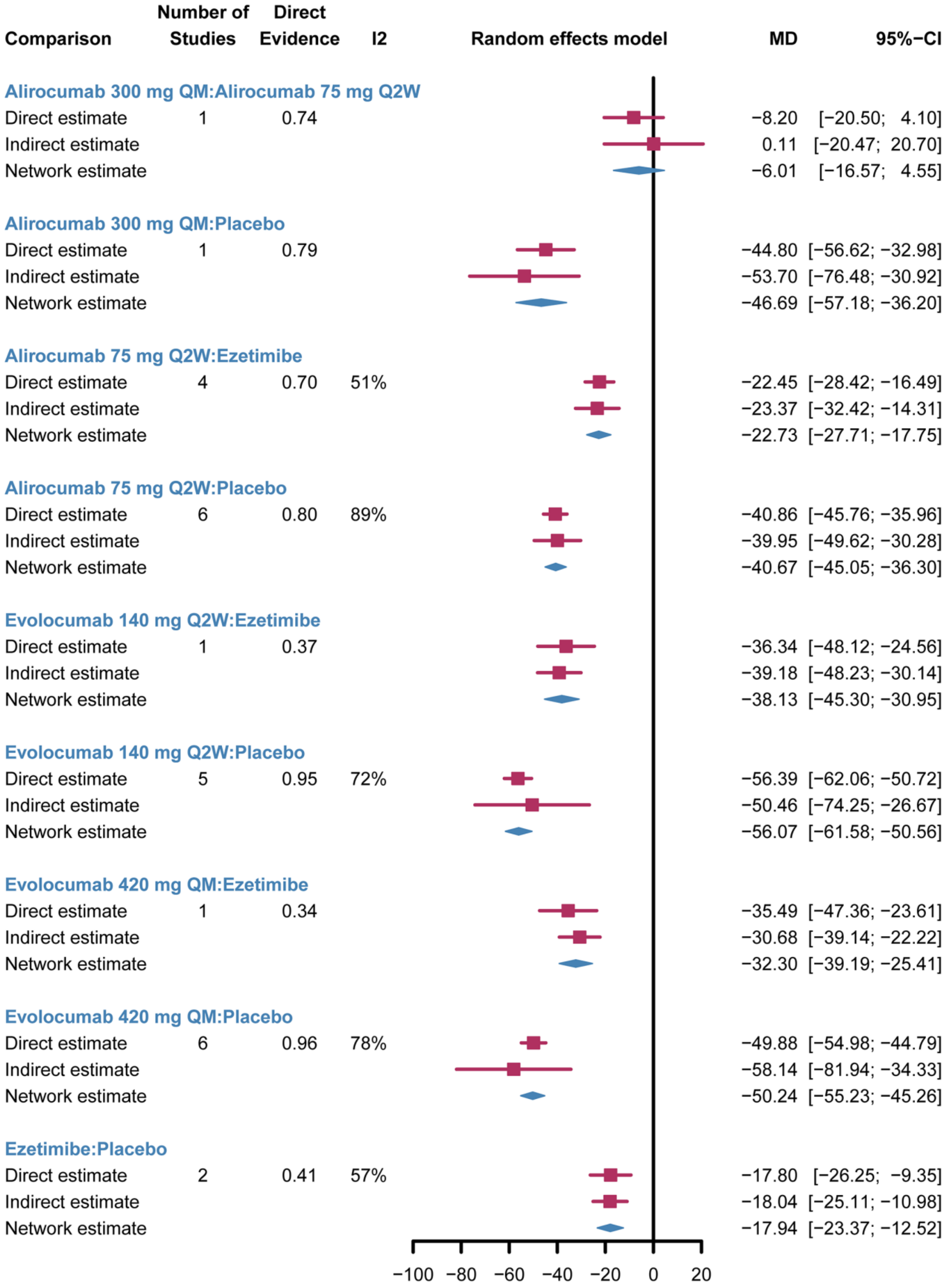

Supplementary Figure S20. Direct and indirect evidence for estimating the percentage change in ApoB (different drug dose).

Comparison	Number of Studies	Direct Evidence	I2	MD	95%−CI
**Alirocumab 300 mg QM:Alirocumab 75 mg Q2W**					
Direct estimate	1	0.74		−7.20	[−22.77; 8.37]
Indirect estimate				4.57	[−21.40; 30.54]
Network estimate				−4.09	[−17.44; 9.27]
**Alirocumab 300 mg QM:Placebo**					
Direct estimate	1	0.79		−26.60	[−41.55; −11.65]
Indirect estimate				−39.21	[−68.00; −10.42]
Network estimate				−29.28	[−42.55; −16.01]
**Alirocumab 75 mg Q2W:Ezetimibe**					
Direct estimate	4	0.69	85%	−26.84	[−34.48; −19.19]
Indirect estimate				−24.59	[−36.10; −13.08]
Network estimate				−26.15	[−32.52; −19.78]
**Alirocumab 75 mg Q2W:Placebo**					
Direct estimate	6	0.80	91%	−24.73	[−30.97; −18.49]
Indirect estimate				−26.98	[−39.31; −14.66]
Network estimate				−25.19	[−30.76; −19.62]
**Evolocumab 140 mg Q2W:Ezetimibe**					
Direct estimate	1	0.42		−33.62	[−48.45; −18.80]
Indirect estimate				−43.83	[−56.51; −31.14]
Network estimate				−39.51	[−49.15; −29.88]
**Evolocumab 140 mg Q2W:Placebo**					
Direct estimate	5	0.93	71%	−39.11	[−47.32; −30.89]
Indirect estimate				−31.24	[−61.18; −1.29]
Network estimate				−38.56	[−46.48; −30.64]
**Evolocumab 420 mg QM:Ezetimibe**					
Direct estimate	1	0.37		−32.10	[−47.07; −17.12]
Indirect estimate				−36.42	[−47.83; −25.00]
Network estimate				−34.83	[−43.91; −25.75]
**Evolocumab 420 mg QM:Placebo**					
Direct estimate	6	0.95	60%	−33.76	[−40.89; −26.64]
Indirect estimate				−35.82	[−65.81; −5.82]
Network estimate				−33.87	[−40.80; −26.94]
**Ezetimibe:Placebo**					
Direct estimate	2	0.42	0%	3.43	[−7.23; 14.08]
Indirect estimate				−0.83	[−9.90; 8.24]
Network estimate				0.96	[−5.95; 7.86]

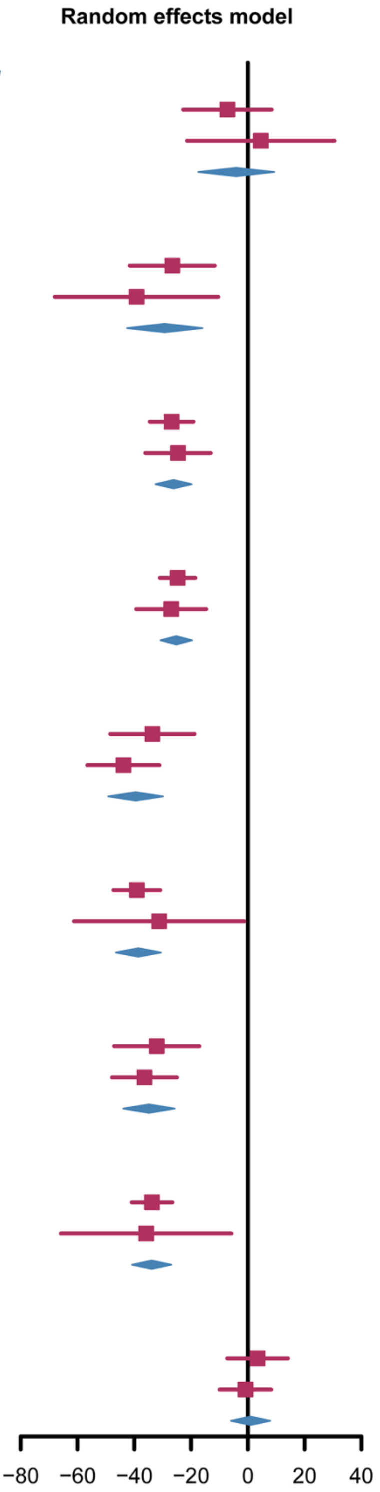

Supplementary Figure S21. Direct and indirect evidence for estimating the percentage change in Lp(a) (different drug dose).

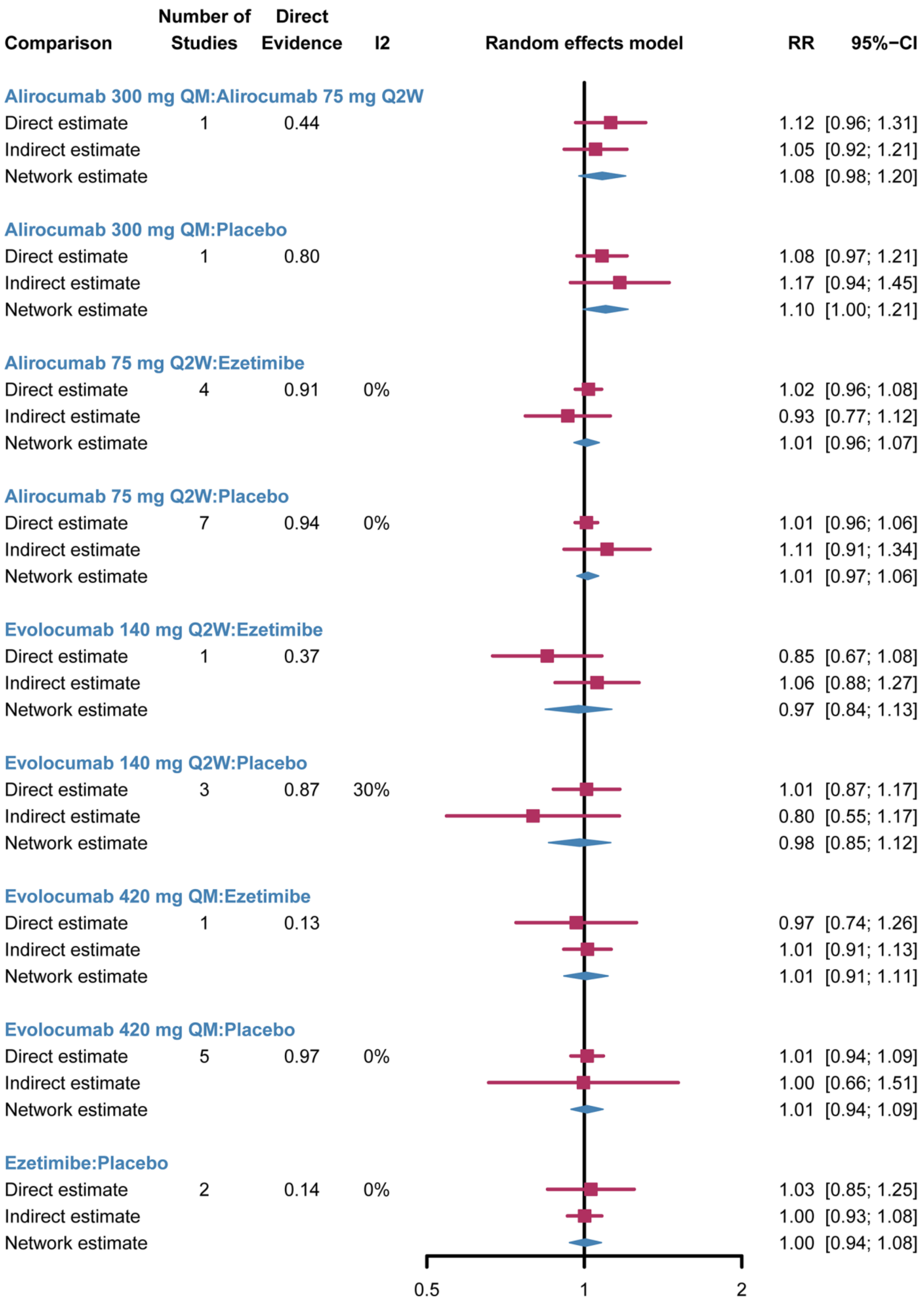

Supplementary Figure S22. Direct and indirect evidence for estimating the risk ratio of AE (different drug dose).

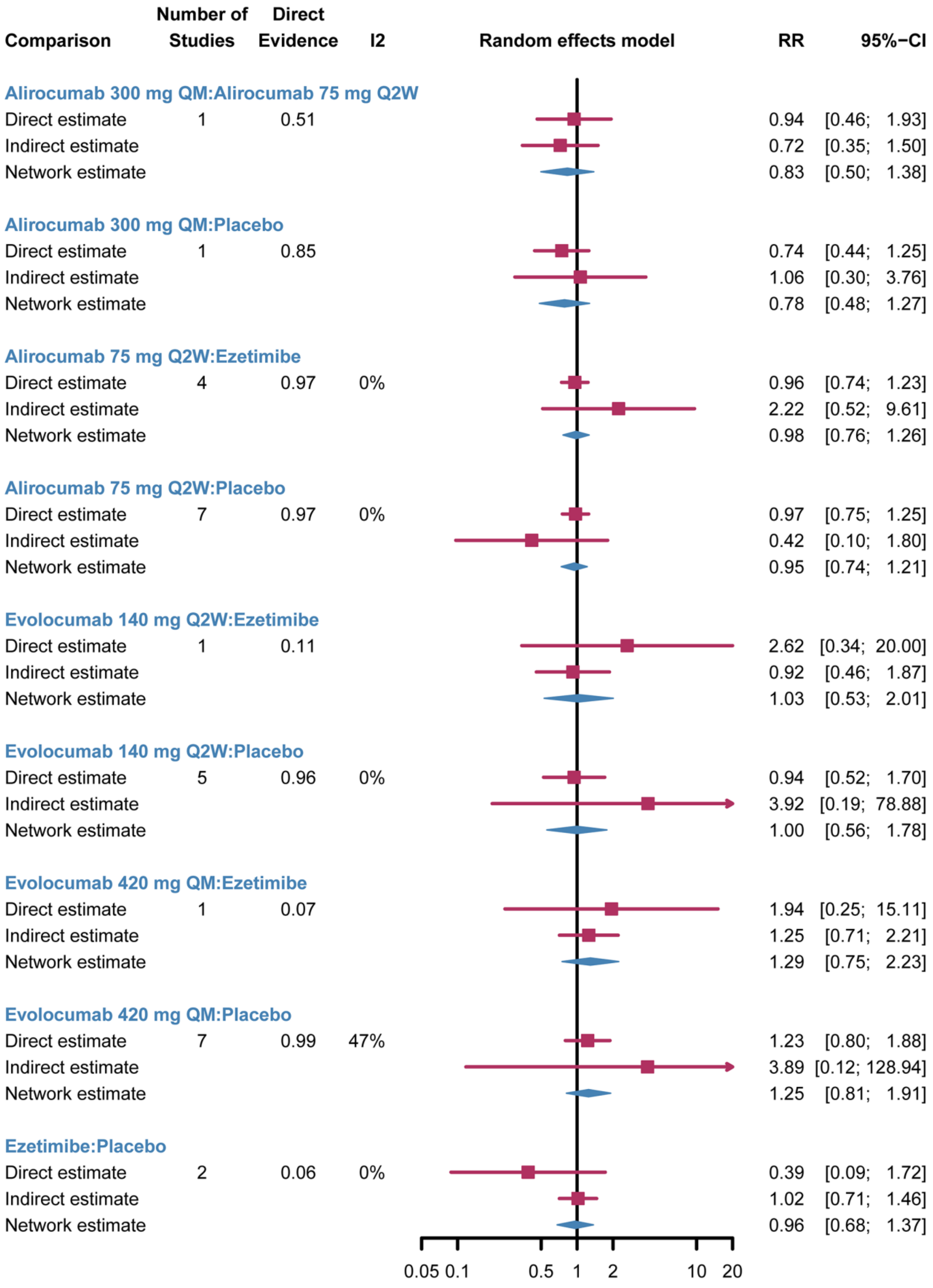

Supplementary Figure S23. Direct and indirect evidence for estimating the risk ratio of SAE (different drug dose).

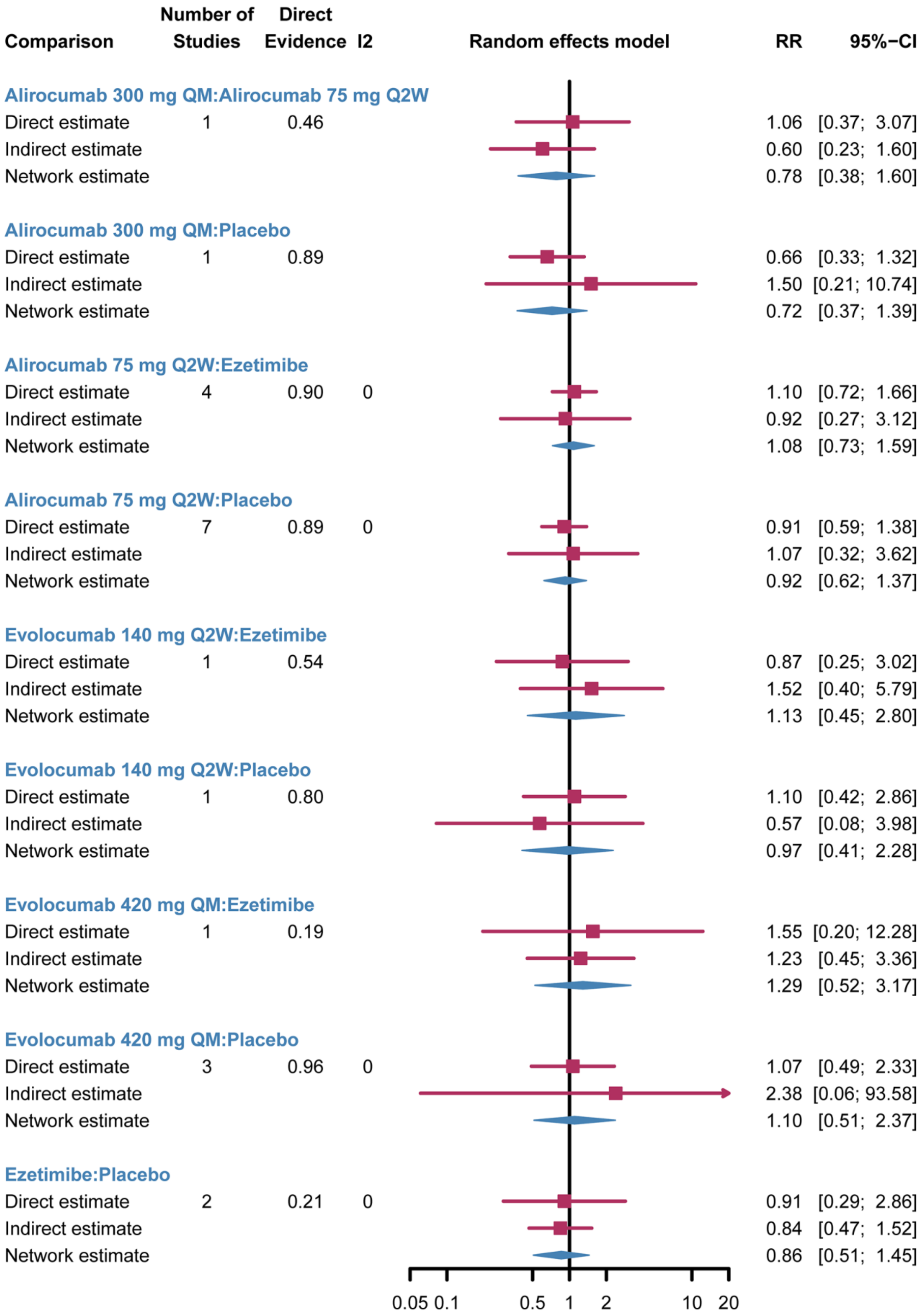

Supplementary Figure S24. Direct and indirect evidence for estimating the risk ratio of AE leading to treatment discontinuation (different drug dose).

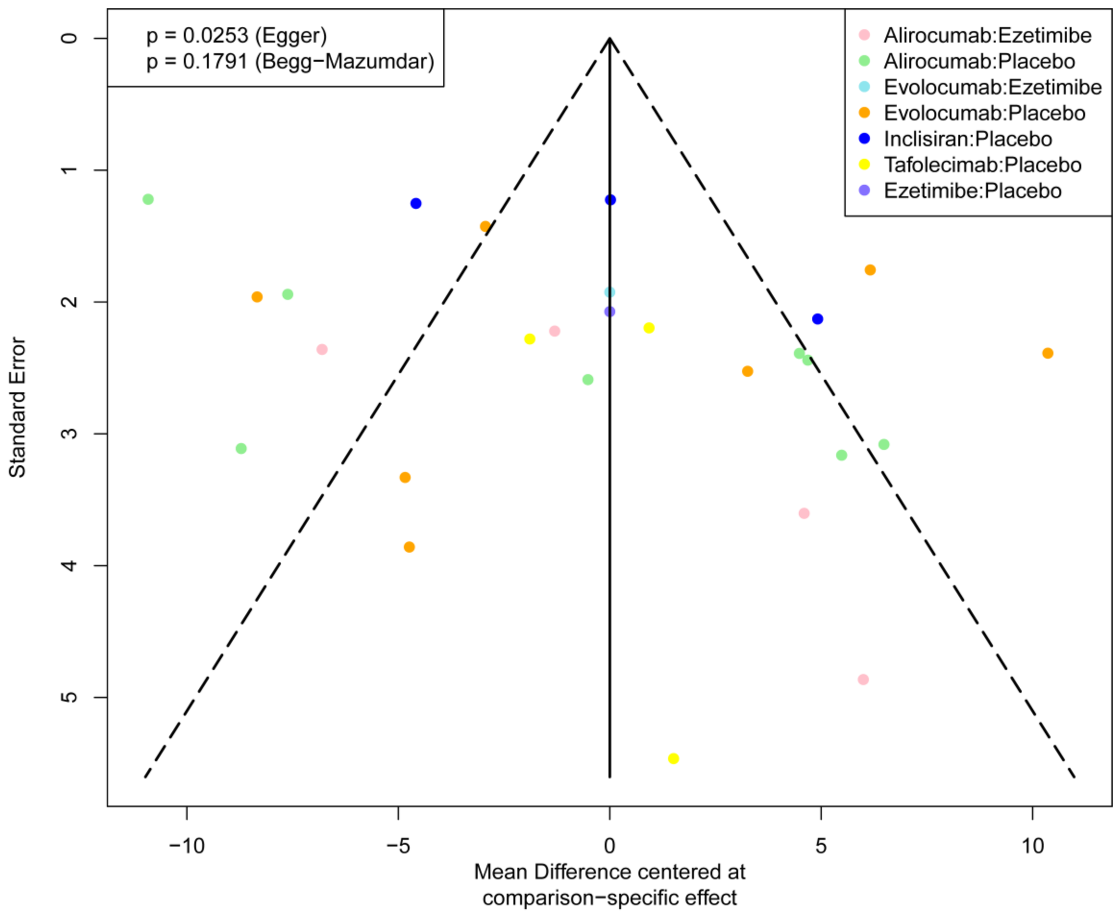

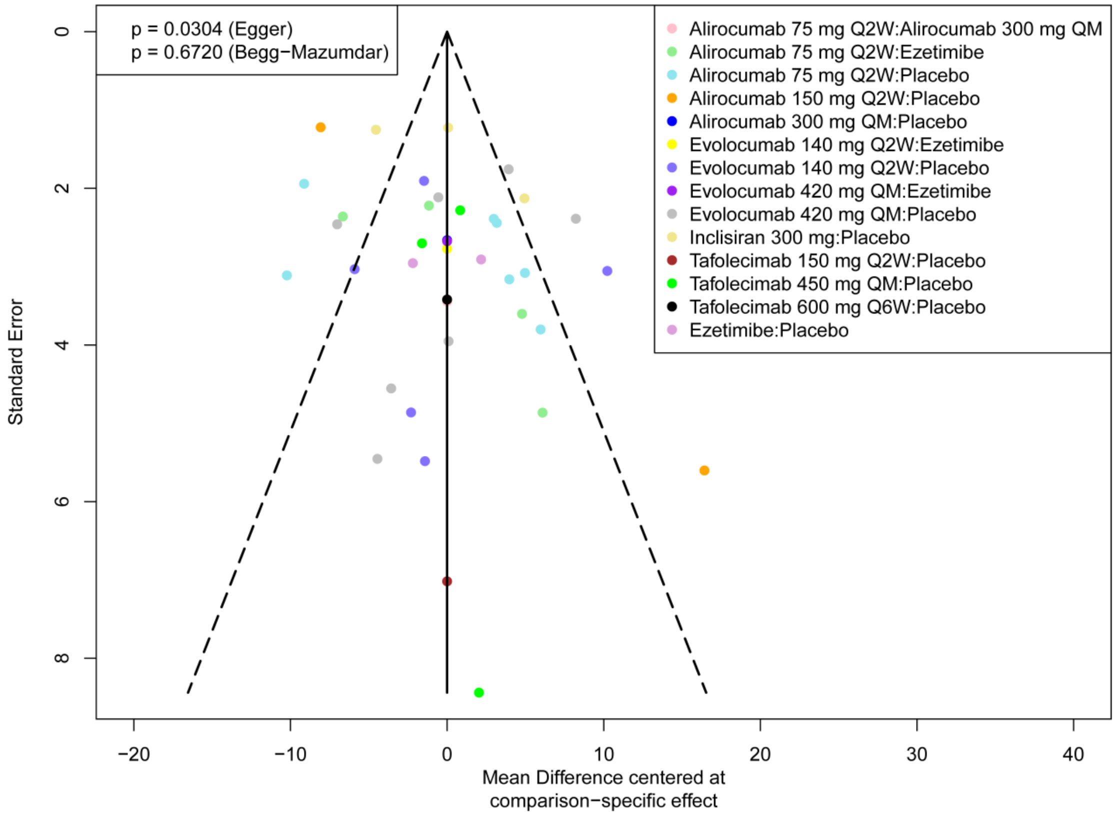

Supplementary Figure S25. Funnel plot for LDL-C.

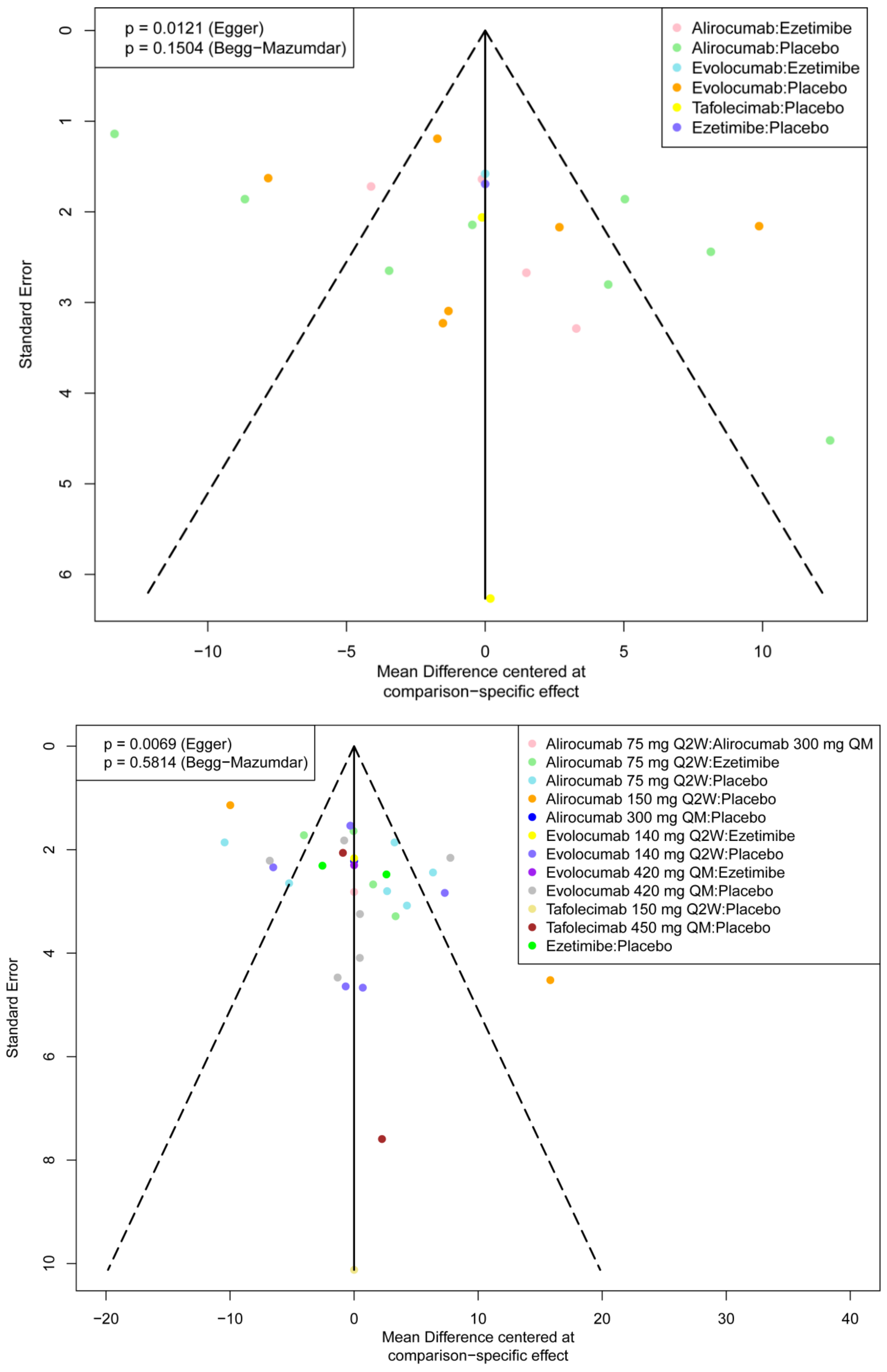

Supplementary Figure S26. Funnel plot for ApoB.

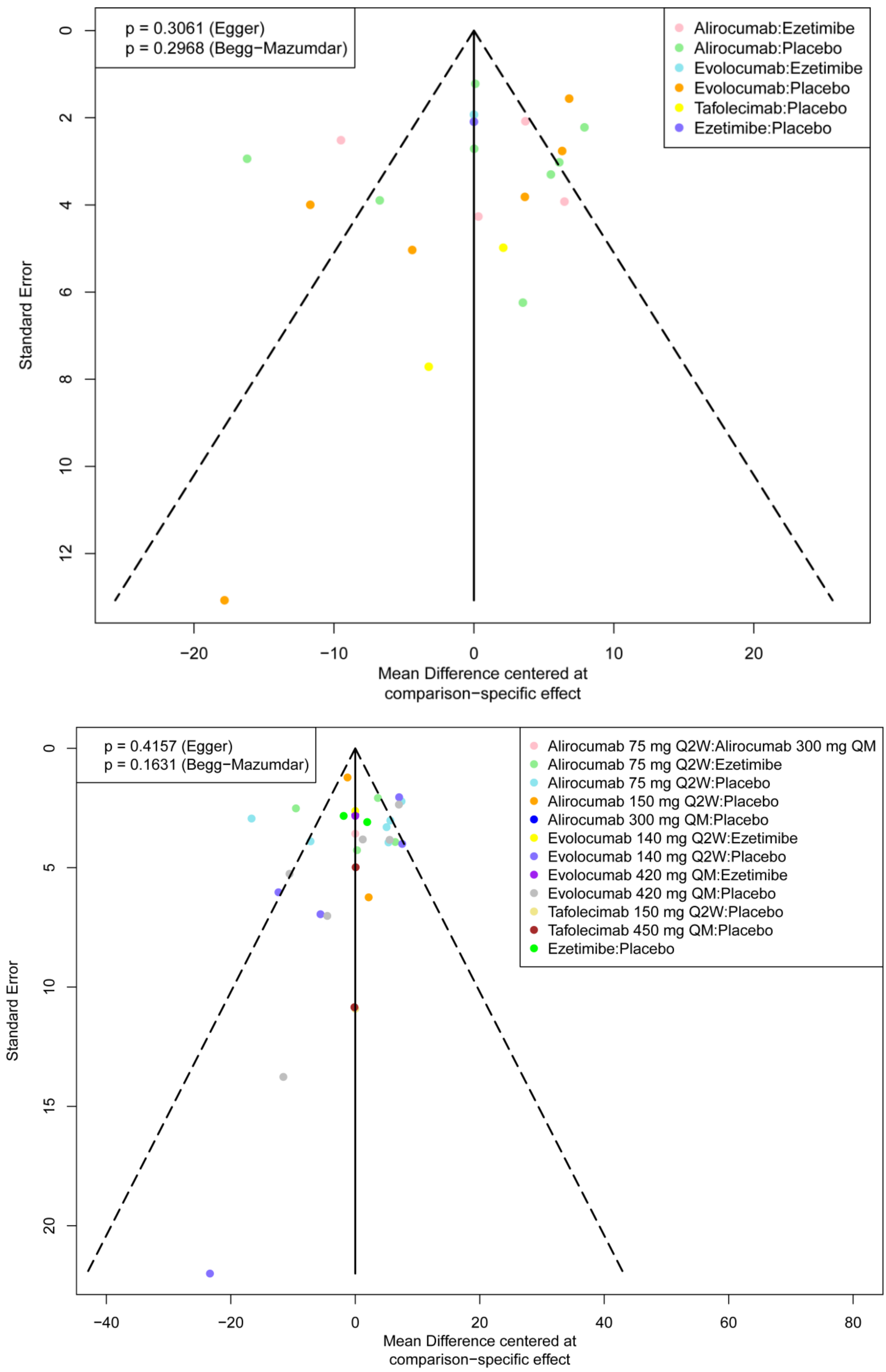

Supplementary Figure S27. Funnel plot for Lp(a).

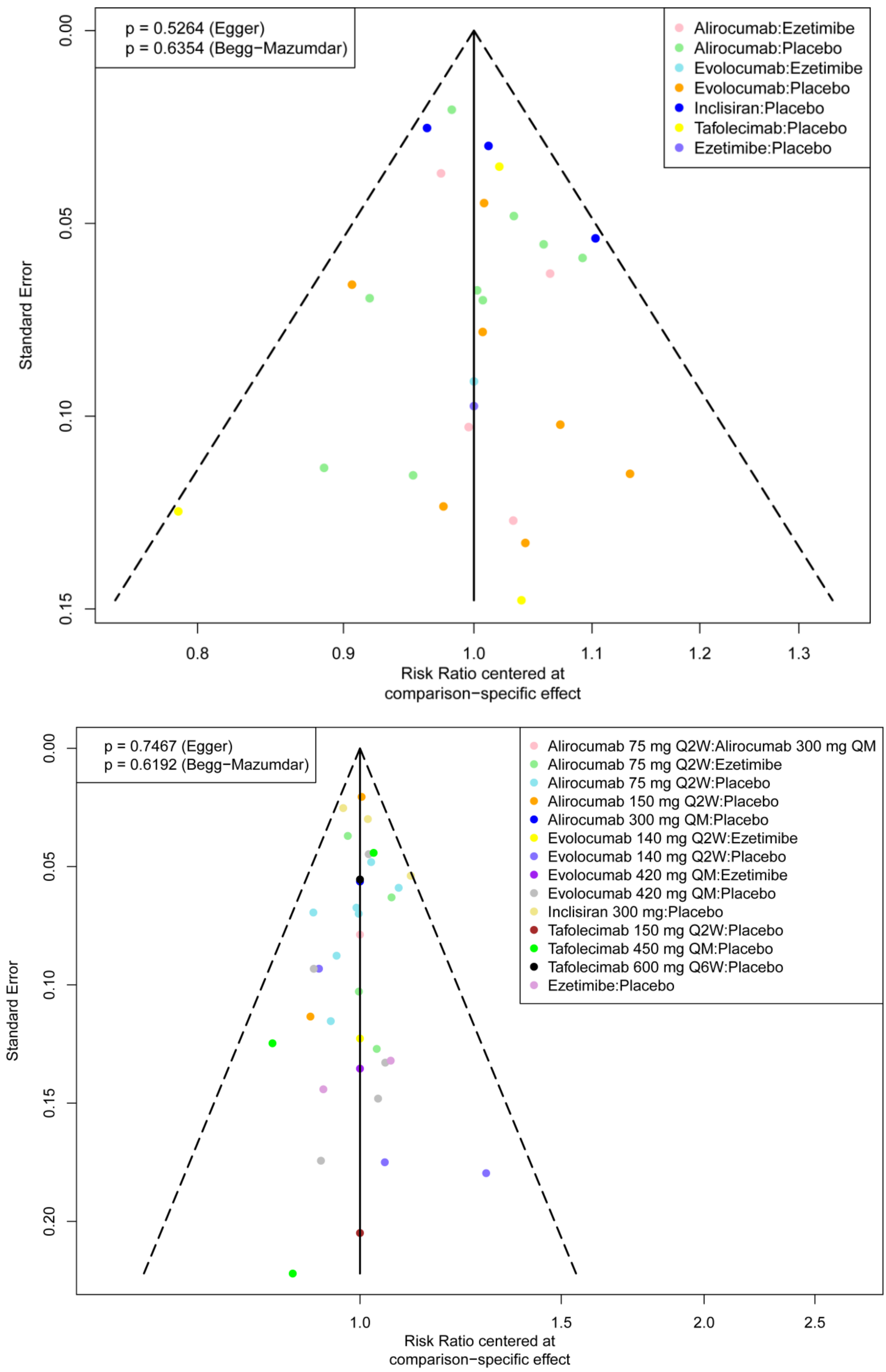

Supplementary Figure S28. Funnel plot for AE.

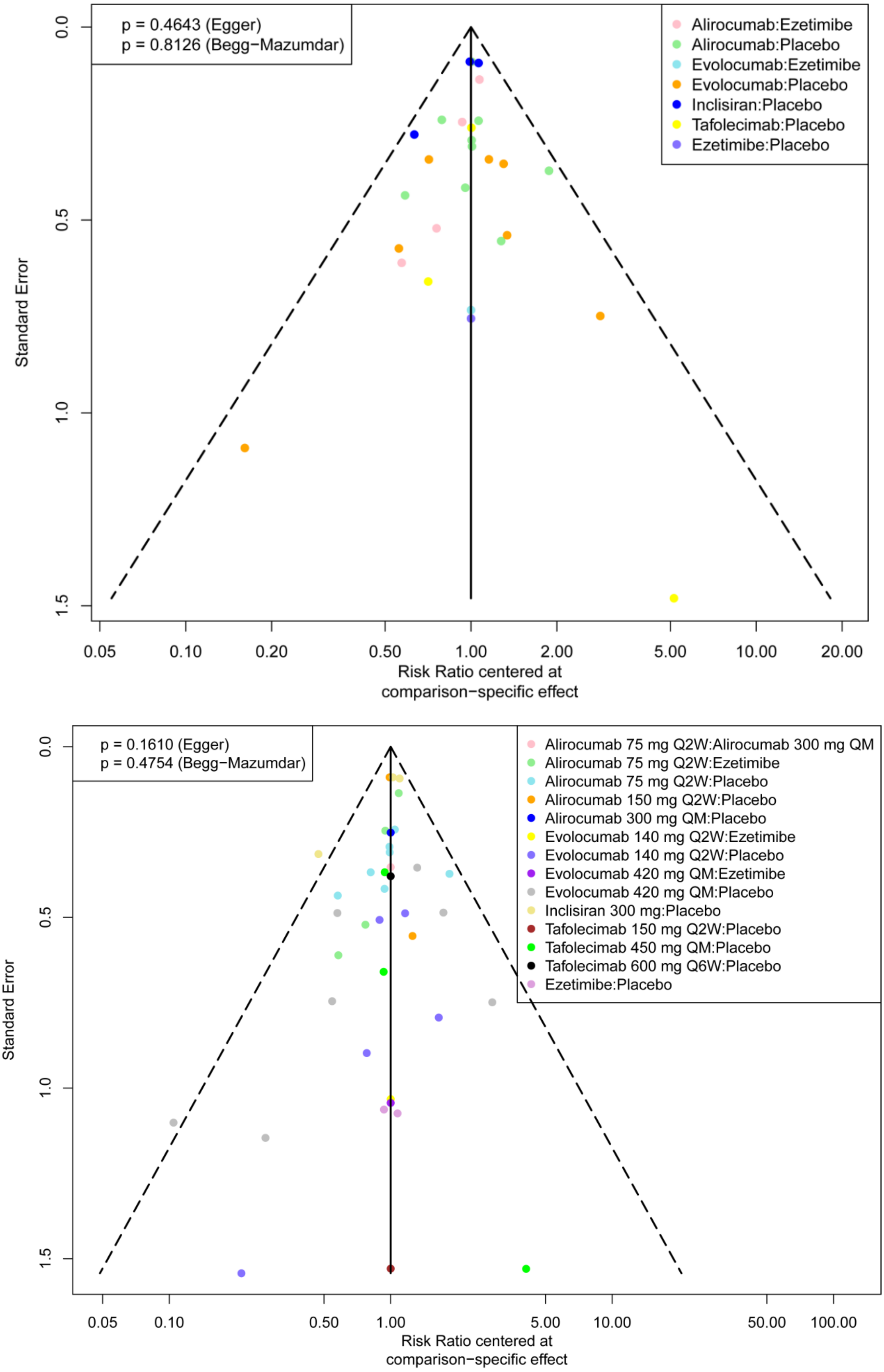

Supplementary Figure S29. Funnel plot for SAE.

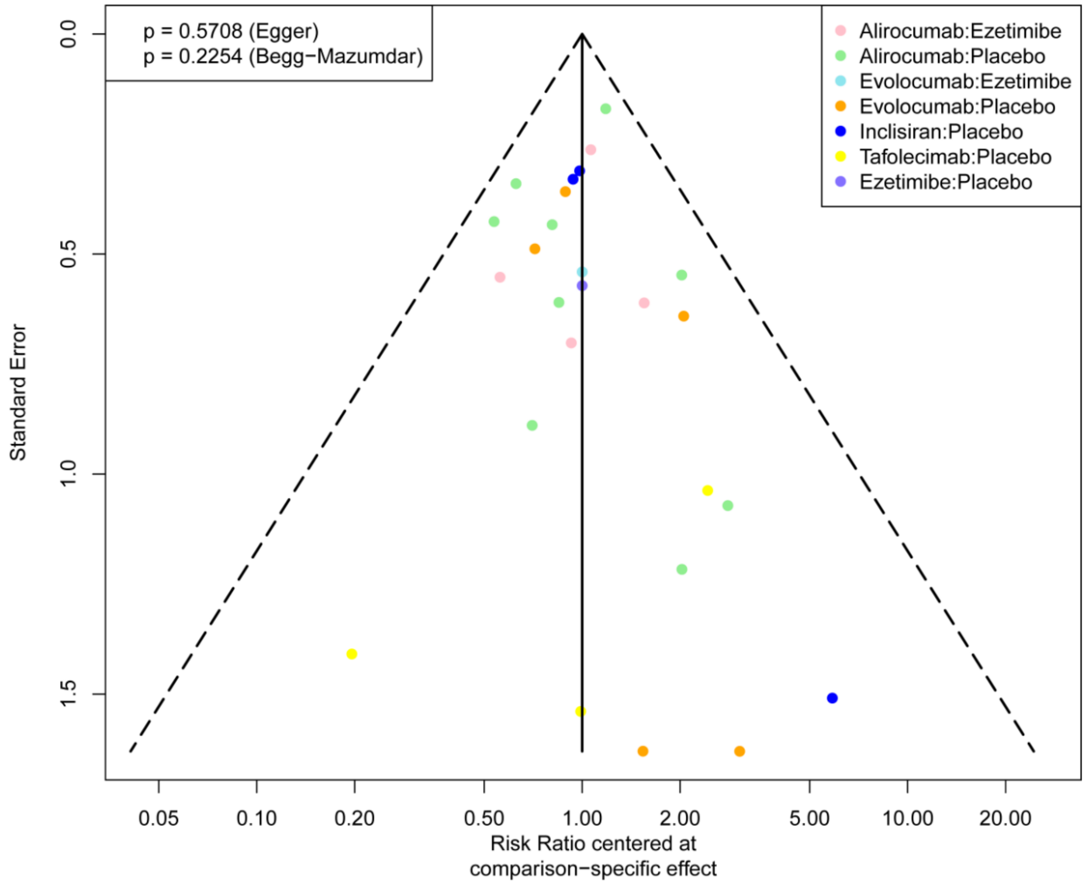

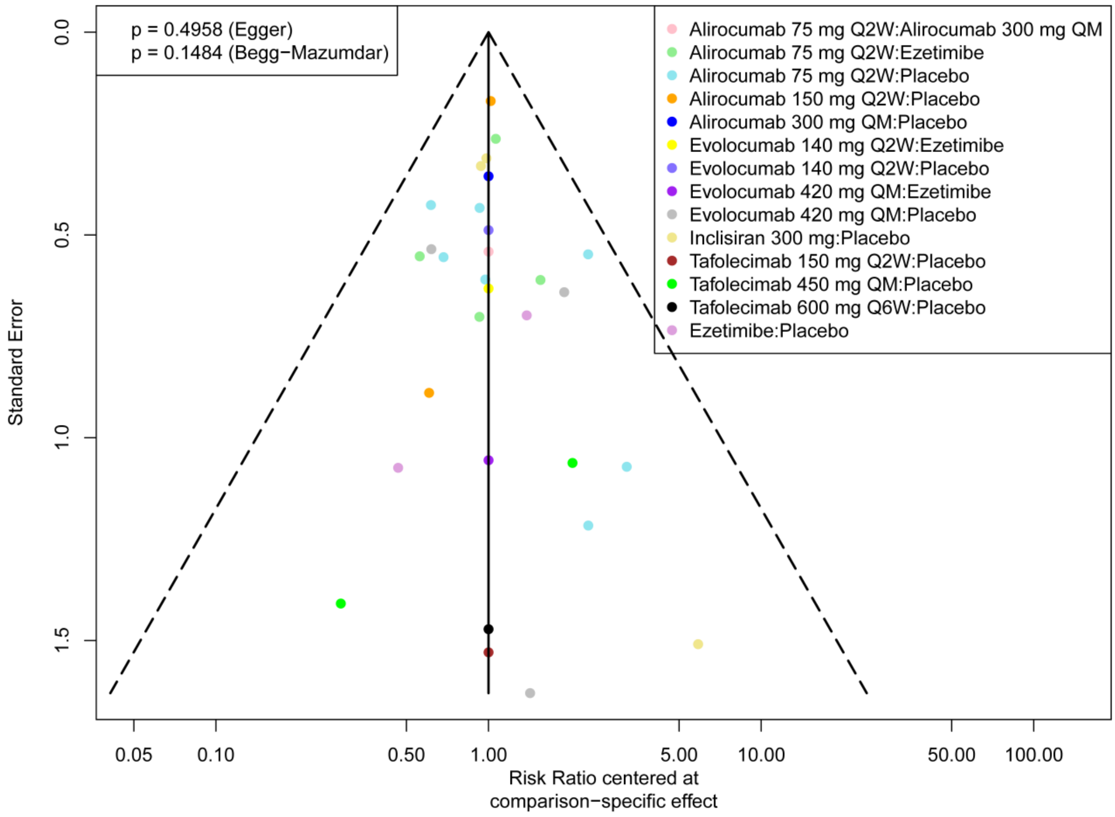

Supplementary Figure S30. Funnel plot for AE leading to treatment discontinuation.

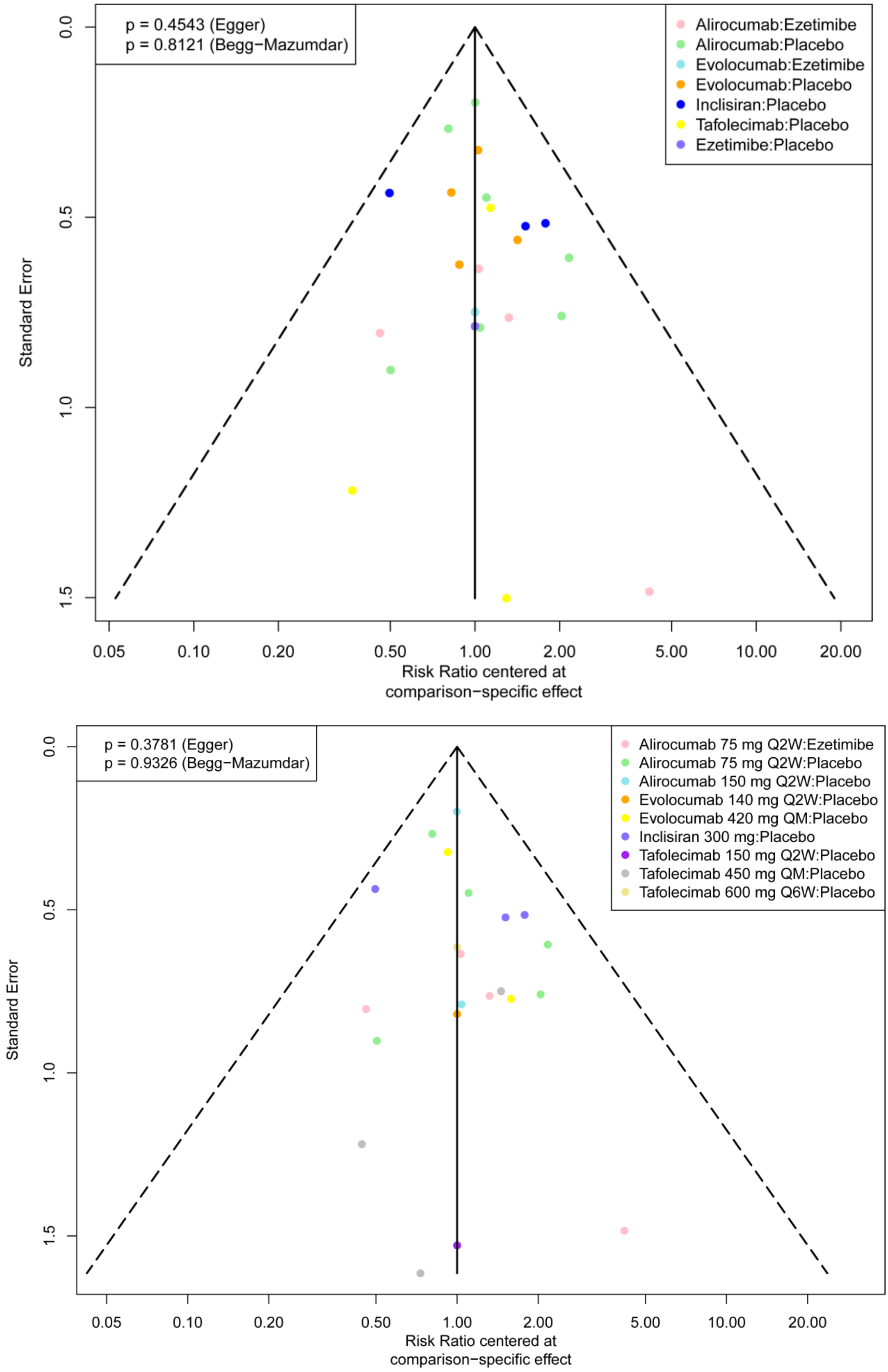

Supplementary Figure S31. Funnel plot for injection-site reaction.

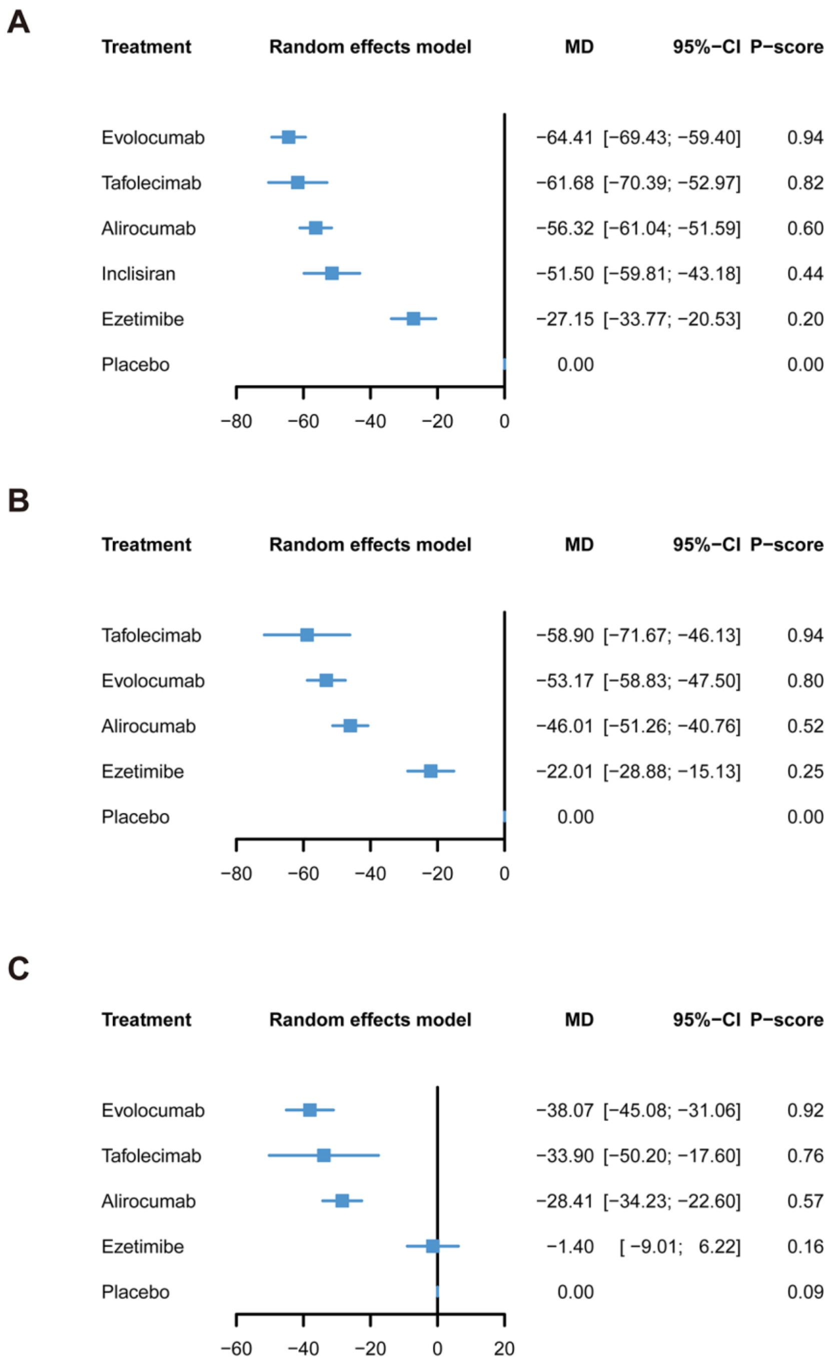

Supplementary Figure S32. Sensitivity analyses for the percentage change in (A) LDL-C; (B) ApoB and (C) Lp(a), excluding trials in FH patients.

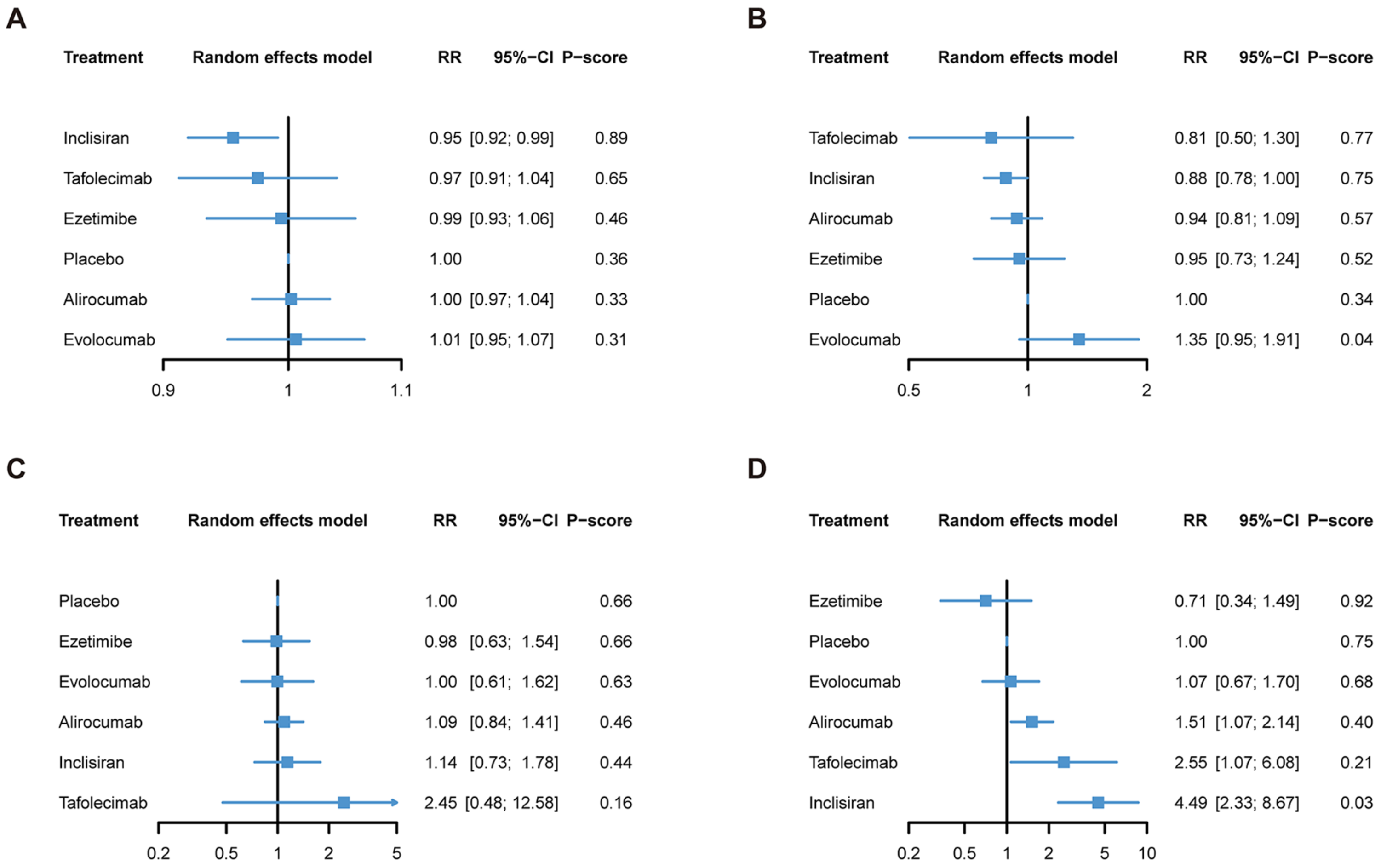

Supplementary Figure S33. Sensitivity analyses for the risk ratio of (A) AE; (B) SAE; (C) AE leading to treatment discontinuation and (D) injection-site reaction, excluding trials in FH patients.

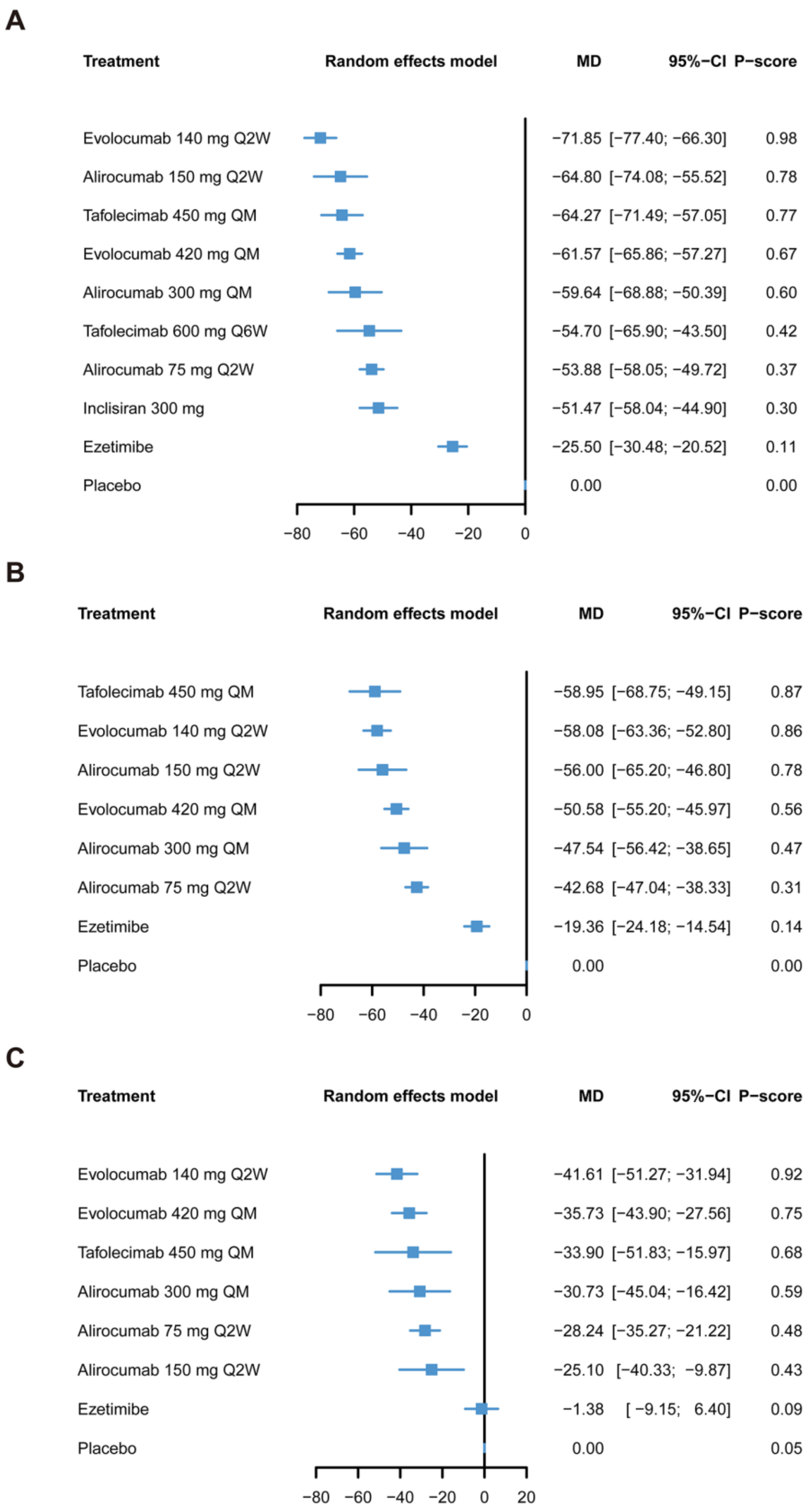

Supplementary Figure S34. Sensitivity analyses for the percentage change in (A) LDL-C; (B) ApoB and (C) Lp(a), excluding trials in FH patients (different drug dose).

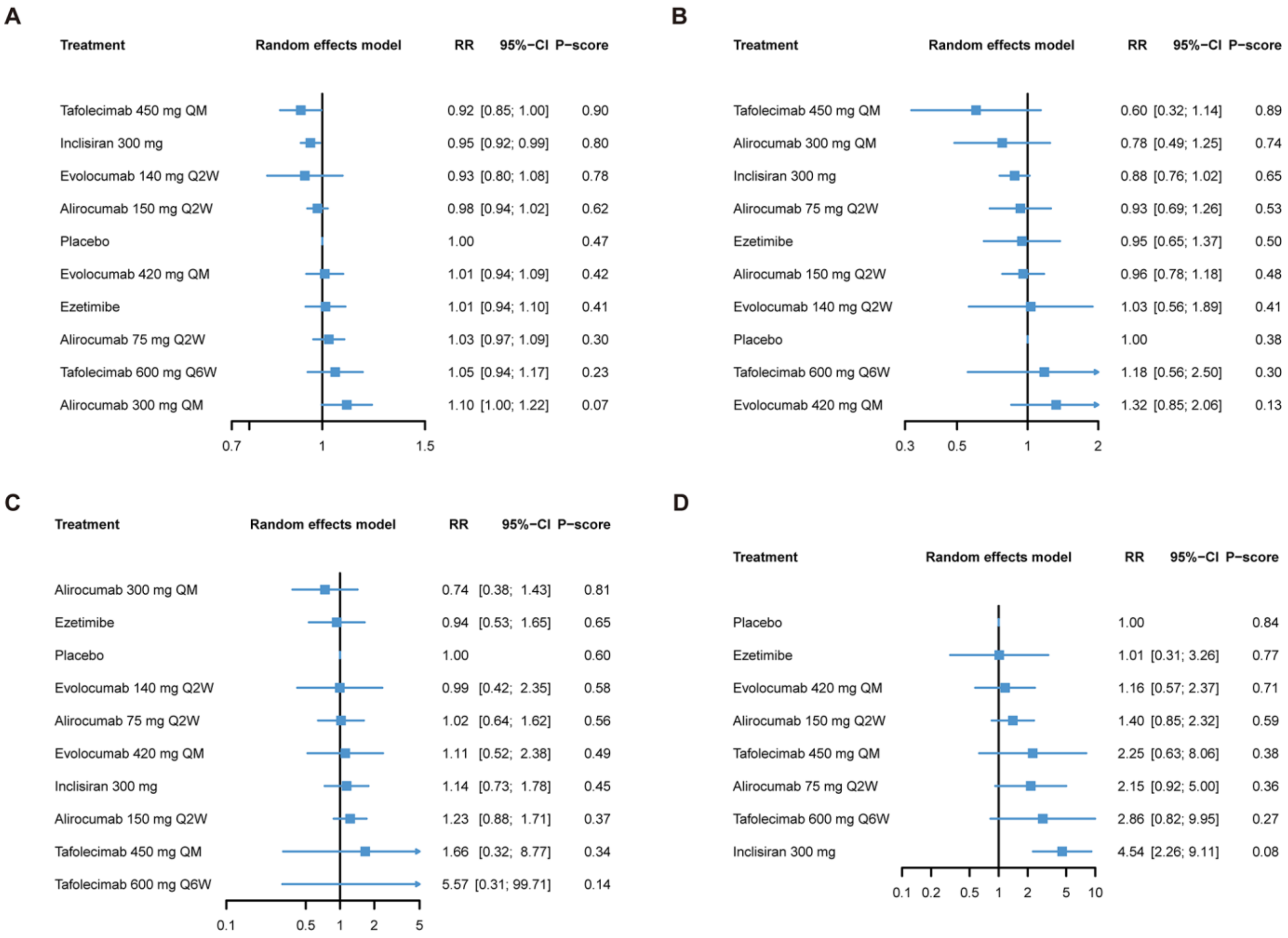

Supplementary Figure S35. Sensitivity analyses for the risk ratio of (A) AE; (B) SAE; (C) AE leading to treatment discontinuation and (D) injection-site reaction, excluding trials in FH patients (different drug dose).

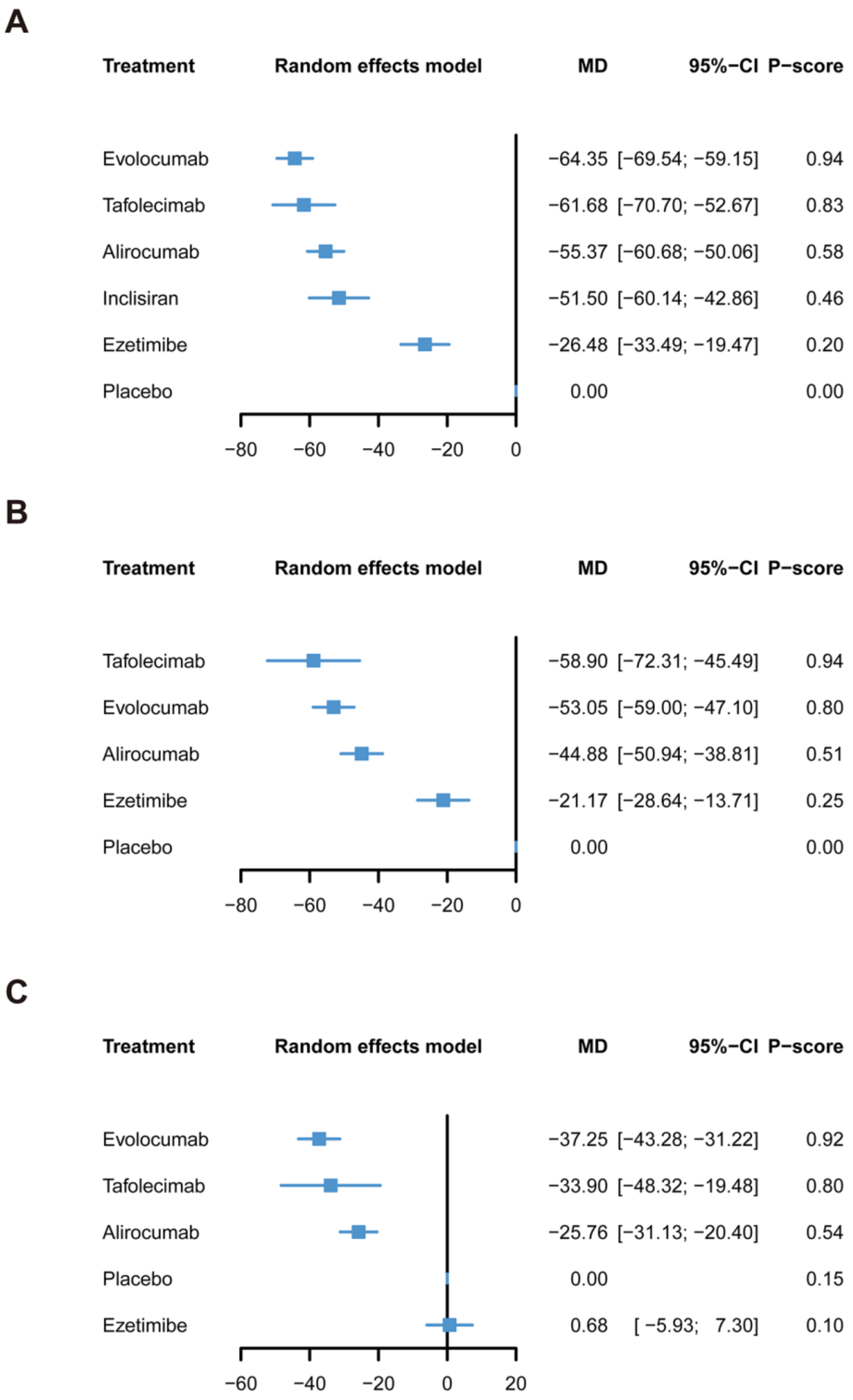

Supplementary Figure S36. Sensitivity analyses for the percentage change in (A) LDL-C; (B) ApoB and (C) Lp(a), excluding baseline LDL-C level > 130 mg/dL.

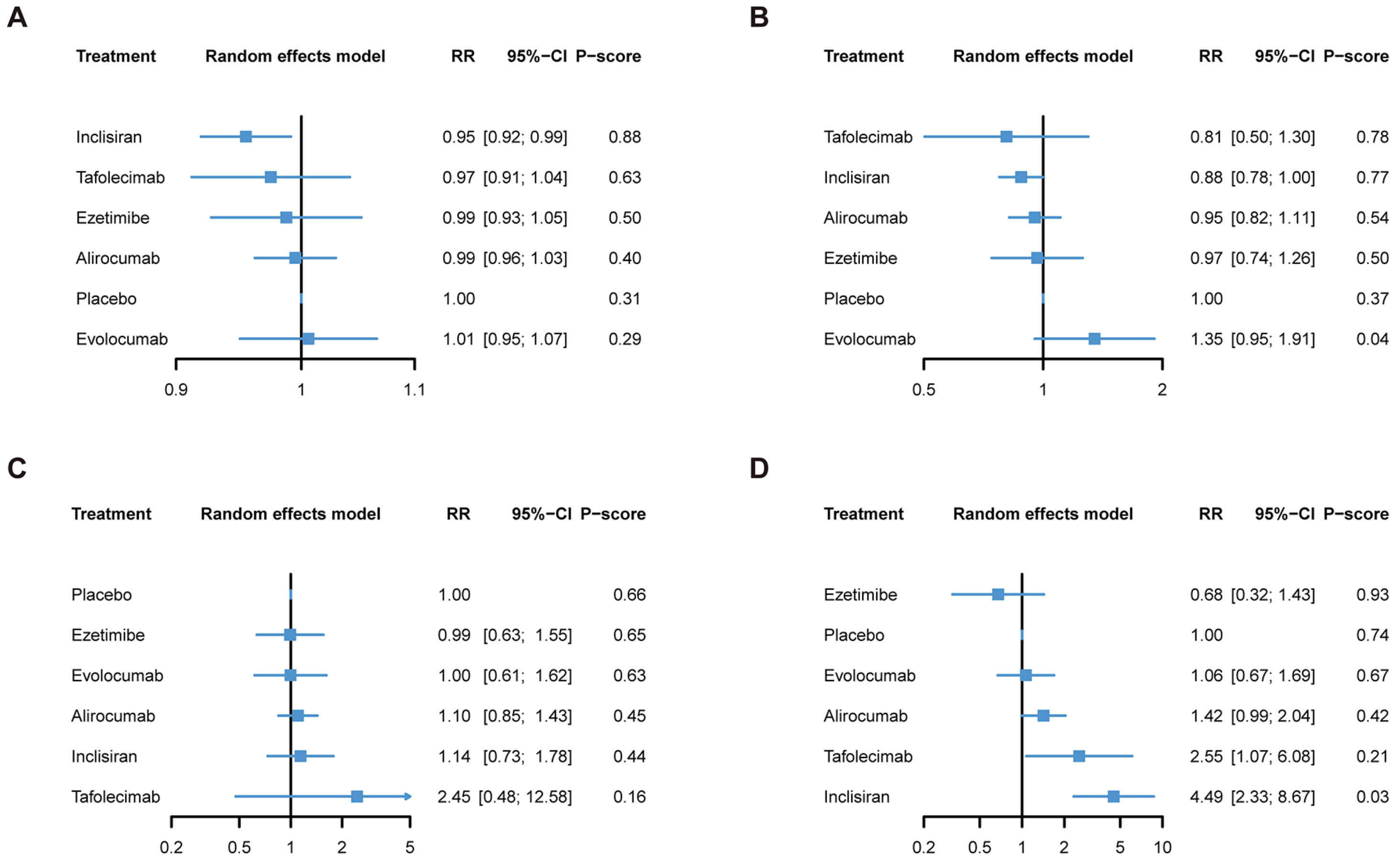

Supplementary Figure S37. Sensitivity analyses for the risk ratio of (A) AE; (B) SAE; (C) AE leading to treatment discontinuation and (D) injection-site reaction, excluding baseline LDL-C level > 130 mg/dL.

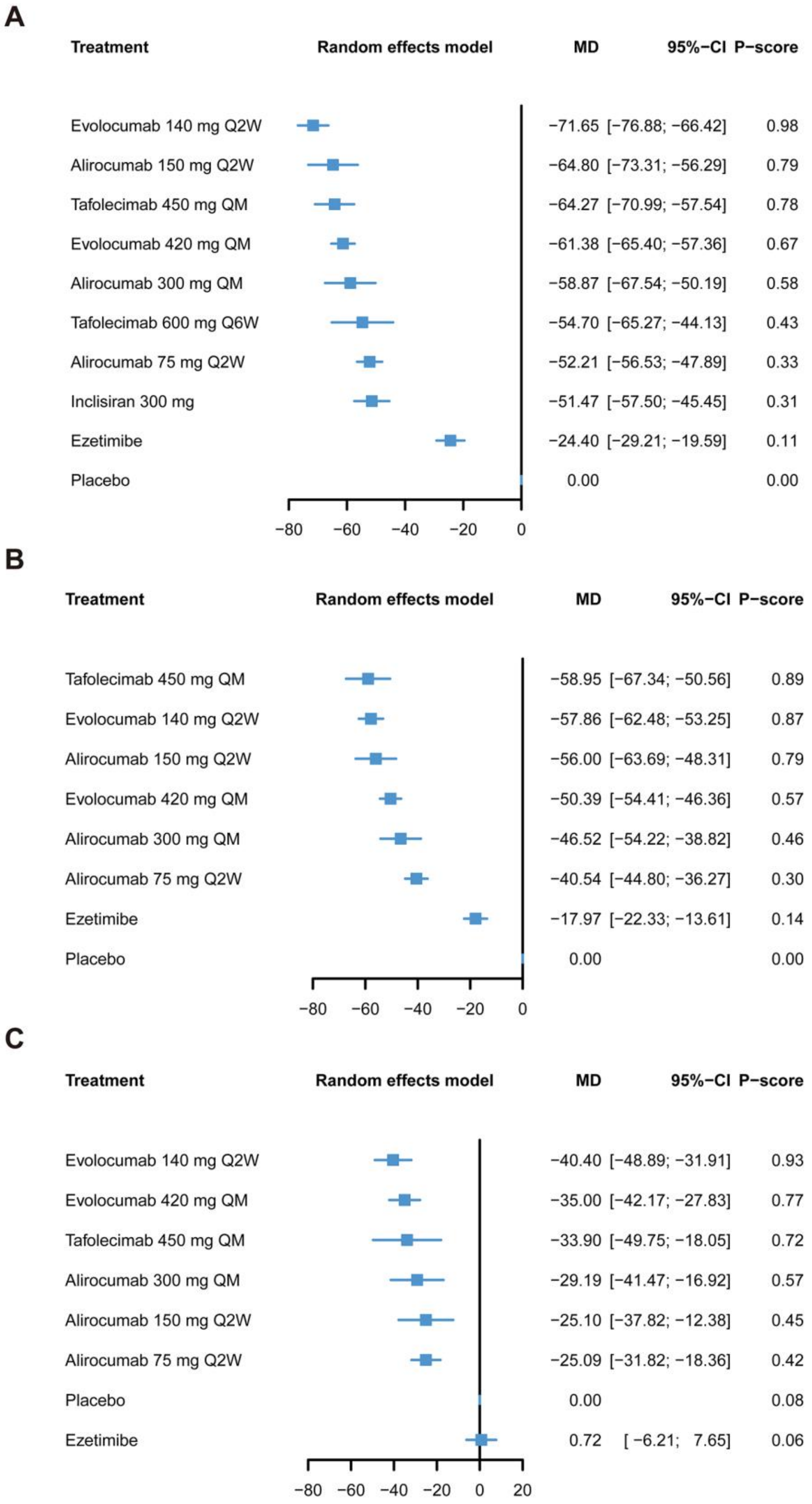

Supplementary Figure S38. Sensitivity analyses for the percentage change in (A) LDL-C; (B) ApoB and (C) Lp(a), excluding baseline LDL-C level > 130 mg/dL (different drug dose).

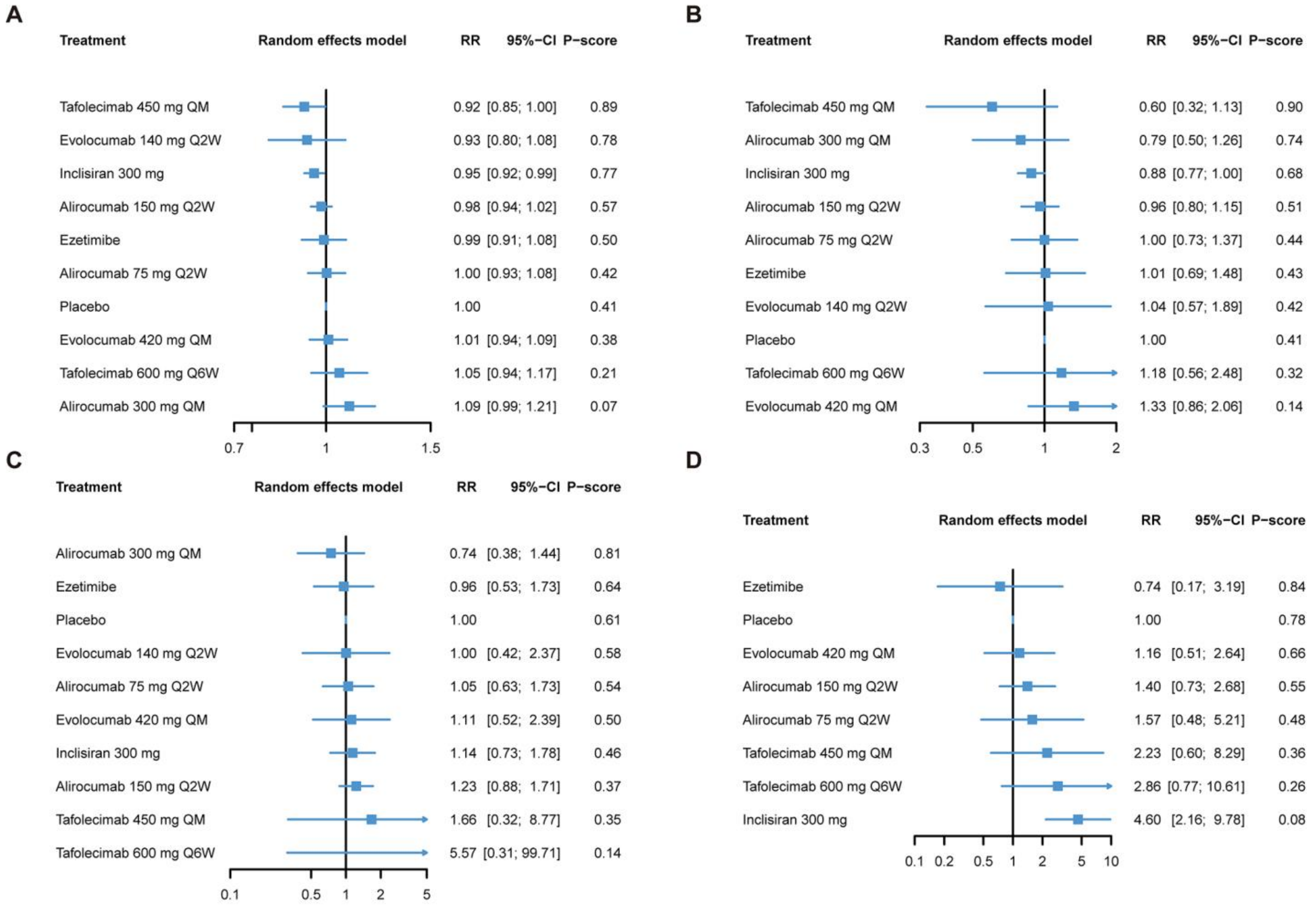

Supplementary Figure S39. Sensitivity analyses for the risk ratio of (A) AE; (B) SAE; (C) AE leading to treatment discontinuation and (D) injection-site reaction, excluding baseline LDL-C level > 130 mg/dL (different drug dose).

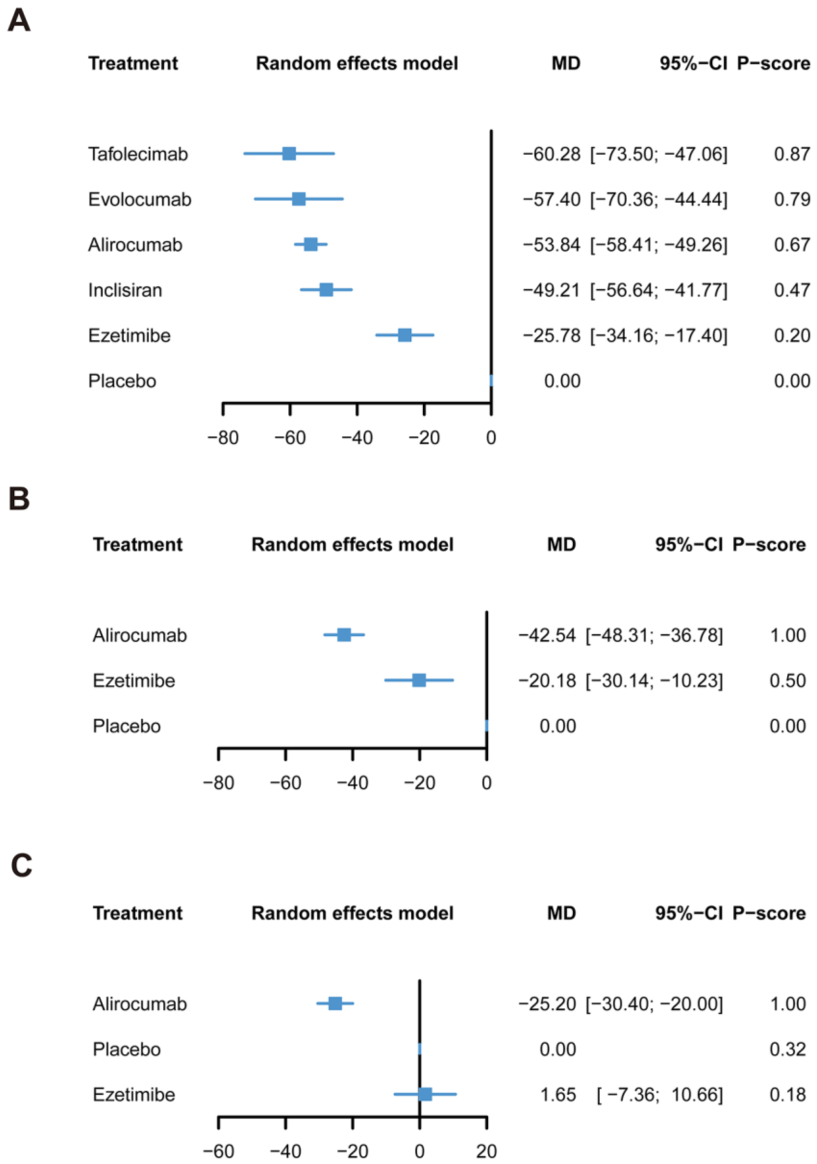

Supplementary Figure S40. Sensitivity analyses for the percentage change in (A) LDL-C; (B) ApoB and (C) Lp(a), excluding follow-up duration < 24 weeks.

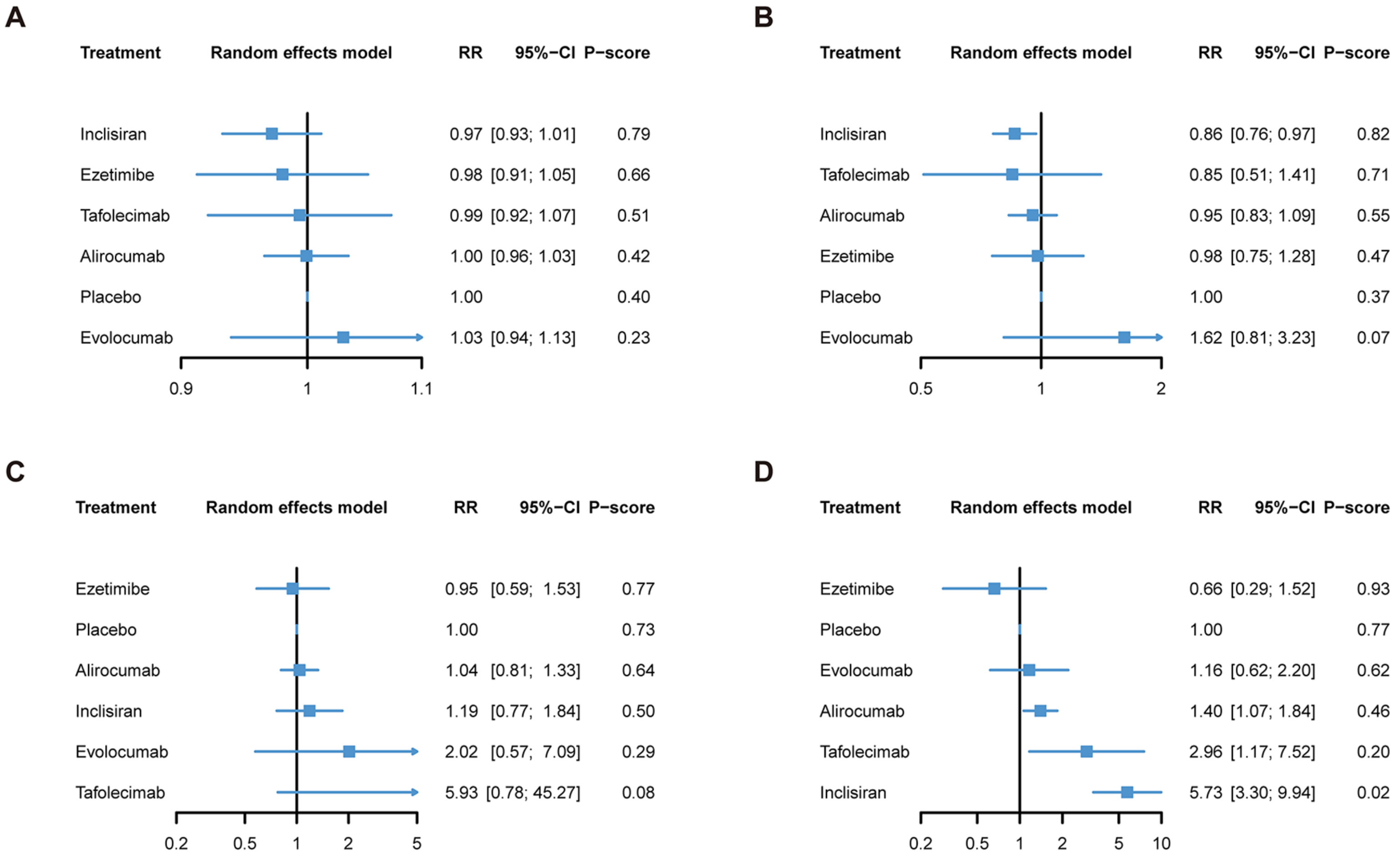

Supplementary Figure S41. Sensitivity analyses for the risk ratio of (A) AE; (B) SAE; (C) AE leading to treatment discontinuation and (D) injection-site reaction, excluding follow-up duration < 24 weeks.

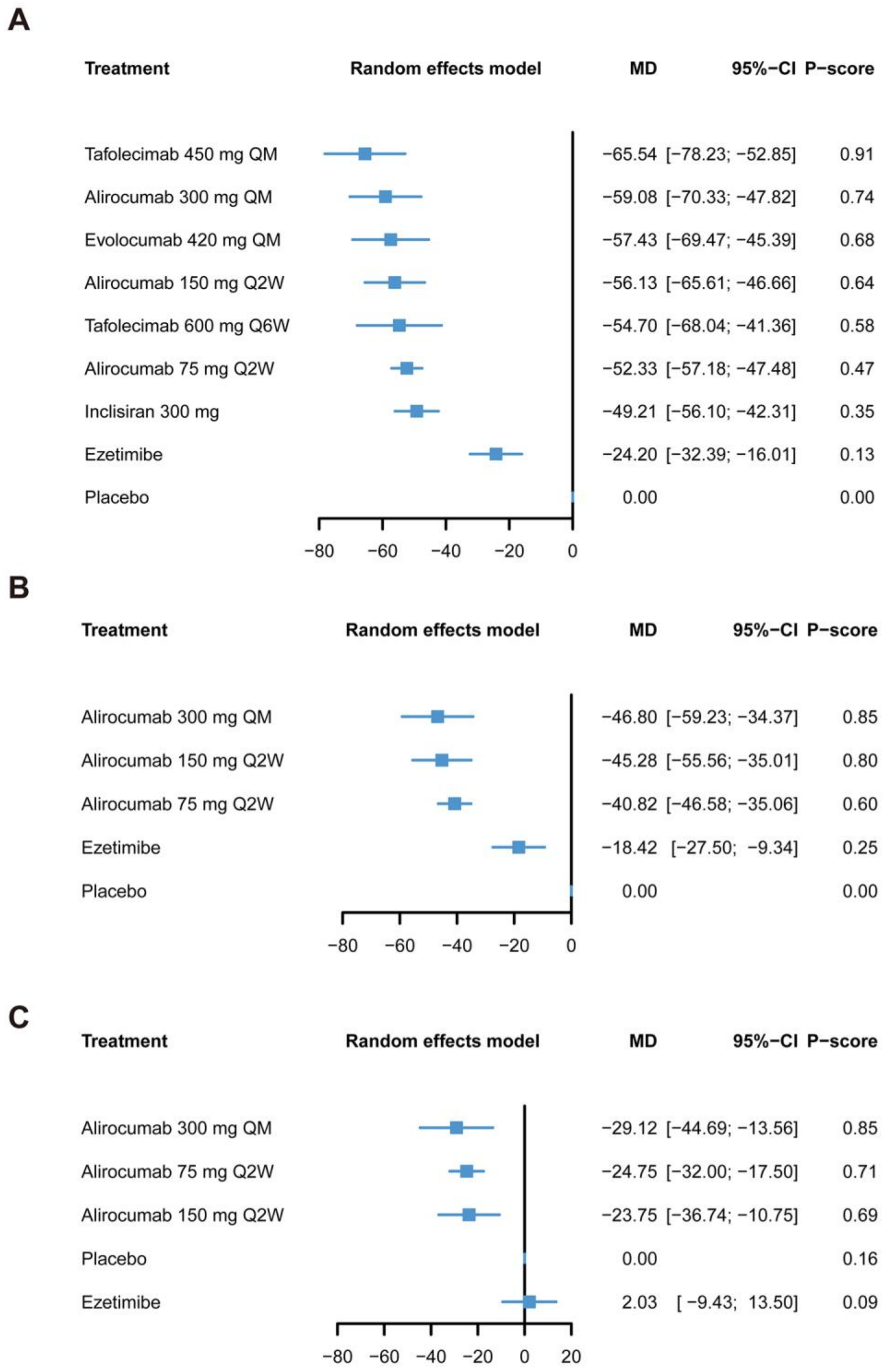

Supplementary Figure S42. Sensitivity analyses for the percentage change in (A) LDL-C; (B) ApoB and (C) Lp(a), excluding follow-up duration < 24 weeks (different drug dose).

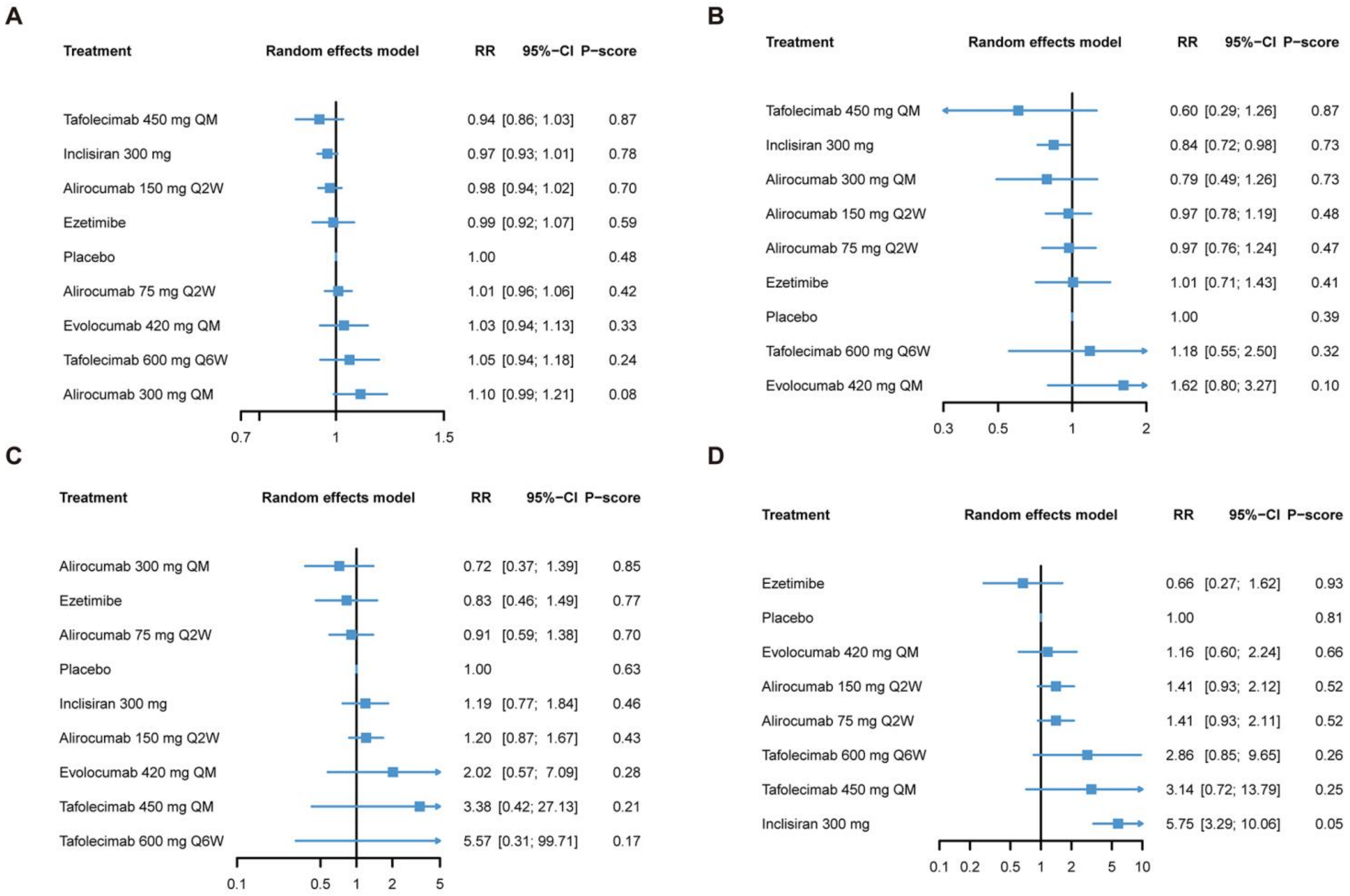

Supplementary Figure S43. Sensitivity analyses for the risk ratio of (A) AE; (B) SAE; (C) AE leading to treatment discontinuation and (D) injection-site reaction, excluding follow-up duration < 24 weeks (different drug dose).

2 Supplementary Tables

Supplementary Table S1. Search strategy (a) Pubmed; (b) Web of science; (c) Embase; (d) Cochrane; (e) ClinicalTrials.gov.

(a) Pubmed

Serial	Search strategy	12 Nov. 2023
#1	((((((((((((("Hypercholesterolemia"[Mesh]) OR (Hypercholesterolemia[Title/Abstract])) OR (Hypercholesterolemias[Title/Abstract])) OR (High Cholesterol Levels[Title/Abstract])) OR (Cholesterol Level, High[Title/Abstract])) OR (Cholesterol Levels, High[Title/Abstract])) OR (High Cholesterol Level[Title/Abstract])) OR (Level, High Cholesterol[Title/Abstract])) OR (Levels, High Cholesterol[Title/Abstract])) OR (Elevated Cholesterol[Title/Abstract])) OR (Cholesterol, Elevated[Title/Abstract])) OR (Cholesterols, Elevated[Title/Abstract])) OR (Elevated Cholesterols[Title/Abstract])) OR (Hypercholesteremia[Title/Abstract])) OR (Hypercholesteremias[Title/Abstract])	70,744
#2	((((((((((((((alirocumab[Title/Abstract]) OR (SAR236553[Title/Abstract])) OR (REGN-727[Title/Abstract])) OR (monoclonal antibody REGN727[Title/Abstract])) OR (REGN727 monoclonal antibody[Title/Abstract])) OR (REGN727[Title/Abstract])) OR (praluent[Title/Abstract])) OR (Evolocumab[Title/Abstract])) OR (repatha[Title/Abstract])) OR (AMG-145[Title/Abstract])) OR (AMG 145[Title/Abstract])) OR (ALN-PCS[Title/Abstract])) OR (leqvio[Title/Abstract])) OR (ALN-PCSsc[Title/Abstract])) OR (Inclisiran[Title/Abstract])) OR (ALN 60212[Title/Abstract])	1,539
#3	(("randomized controlled trial"[Publication Type] OR "controlled clinical trial"[Publication Type] OR "randomized"[Title/Abstract] OR "placebo"[Title/Abstract] OR "drug therapy"[MeSH Subheading] OR "randomly"[Title/Abstract] OR "trial"[Title/Abstract] OR "groups"[Title/Abstract]) NOT ("animals"[MeSH] NOT "humans"[MeSH]))	5,141,769
	#1 AND #2 AND #3	619

(b) Web of science

Serial	Search strategy	12 Nov. 2023
#1	TS=(Hypercholesterolemia) OR AB=(Hypercholesterolemias OR High Cholesterol Levels OR Cholesterol Level, High OR Cholesterol Levels, High OR High Cholesterol Level OR Level, High Cholesterol OR Levels, High Cholesterol OR Elevated Cholesterol OR Cholesterol, Elevated OR Cholesterols, Elevated OR Elevated 1499Cholesterols OR Hypercholesteremia OR Hypercholesteremias)	192,705
#2	TS=(alirocumab OR SAR236553 OR REGN-727 OR monoclonal antibody REGN727 OR REGN727 monoclonal antibody OR REGN727 OR praluent OR evolocumab OR repatha OR AMG-145 OR AMG 145 OR ALN-PCS OR leqvio OR ALN-PCSsc OR Inclisiran OR Tafolecimab)	3,064

#3	TS=(randomised OR randomized OR randomisation OR randomisation OR placebo* OR (random* AND (allocat* OR assign*)) OR (blind* AND (single OR double OR treble OR triple)))	1,955,947
	#1 AND #2 AND #3	845

c) Embase

Serial	Search strategy	12 Nov. 2023
#1	'Hypercholesterolemias'/exp OR 'High Cholesterol Levels'/exp OR 'Cholesterol Level, High'/exp OR 'Cholesterol Levels, High'/exp OR 'High Cholesterol Level'/exp OR 'Level, High Cholesterol'/exp OR 'Levels, High Cholesterol'/exp OR 'Elevated Cholesterol'/exp OR 'Cholesterol, Elevated'/exp OR 'Cholesterols, Elevated'/exp OR 'Elevated Cholesterols'/exp OR 'Hypercholesteremia'/exp OR 'Hypercholesteremias'/exp	89,709
#2	'alirocumab':ab,ti OR 'SAR236553':ab,ti OR 'REGN-727':ab,ti OR 'monoclonal antibody REGN727':ab,ti OR 'REGN727 monoclonal antibody':ab,ti OR 'REGN727':ab,ti OR 'praluent':ab,ti OR 'evolocumab':ab,ti OR 'repatha':ab,ti OR 'AMG-145':ab,ti OR 'AMG 145':ab,ti OR 'ALN-PCS':ab,ti OR 'leqvio':ab,ti OR 'ALN-PCSsc':ab,ti OR 'Inclisiran':ab,ti OR 'Tafolecimab':ab,ti	2,612
#3	'crossover procedure':de OR 'double-blind procedure':de OR 'randomized controlled trial':de OR 'single-blind procedure':de OR (random* OR factorial* OR crossover* OR cross NEXT/1 over* OR placebo* OR doubl* NEAR/1 blind* OR singl* NEAR/1 blind* OR assign* OR allocat* OR volunteer*):de,ab,ti	3,226,663
	#1 AND #2 AND #3	542

(d) Cochrane

Serial	Search strategy	12 Nov. 2023
#1	MeSH descriptor: [Hypercholesterolemia] explode all trees	4,331
#2	(Hypercholesterolemia or Hypercholesterolemias or High Cholesterol Levels or Cholesterol Level, High or Cholesterol Levels, High or High Cholesterol Level or Level, High Cholesterol or Levels, High Cholesterol or Elevated Cholesterol or Cholesterol, Elevated or Cholesterols, Elevated or Elevated Cholesterols or Hypercholesteremia or Hypercholesteremias):ti,ab,kw	22,527
#3	#1 OR #2	22,527
#4	(alirocumab OR SAR236553 OR REGN-727 OR monoclonal antibody REGN727 OR REGN727 monoclonal antibody OR REGN727 OR praluent OR evolocumab OR repatha OR AMG-145 OR AMG 145 OR ALN-PCS OR leqvio OR ALN-PCSsc OR Inclisiran OR Tafolecimab):ti,ab,kw	1,029
	#3 AND #4	616

(e) Clinicaltrials.gov

Search strategy	Filters	12 Nov. 2023
Recruitment: All studies<br>Study type: Interventional studies (Clinical trials)<br>Study Results: All studies<br>Conditions: Hypercholesterolemia<br>Interventions: alirocumab OR SAR236553 OR REGN-727 OR monoclonal antibody REGN727 OR REGN727 monoclonal antibody OR REGN727 OR praluent OR evolocumab OR repatha OR AMG-145 OR AMG 145 OR ALN-PCS OR leqvio OR ALN-PCSsc OR Inclisiran OR Tafolecimab<br>Study phase: phase 3<br>Eligibility criteria: adult (>=18)	With Results	62

Supplementary Table S3. Results of inconsistency testing.

Outcomes	Within designs	Between designs	Between designs (interaction random effect model)
Treatment			
LDL-C	<0.001	0.010	0.864
ApoB	<0.001	0.004	0.904
Lp(a)	<0.001	0.021	0.514
AE	0.471	0.130	0.130
SAE	0.578	0.330	0.330
AE leading to treatment discontinuation	0.613	0.891	0.891
Injection-site reaction	0.725	0.872	0.872
Treatment with different drug dose			
LDL-C	<0.001	0.231	0.949
ApoB	<0.001	0.233	0.978
Lp(a)	<0.001	0.038	0.756
AE	0.535	0.414	0.414
SAE	0.264	0.638	0.661
AE leading to treatment discontinuation	0.697	0.582	0.582
Injection-site reaction	0.579	-	-

The table shows overall test for inconsistency models. If the p-value is not statistically significant, the overall inconsistency is low.
